# Supplementary material for: LoGicAl: local ancestry and genotype calling uncertainty-aware ancestry-specific allele frequency estimation from admixed samples
Source: Bioinformatics. 2026 Oct 10;42(10):btag752. doi: 10.1093/bioinformatics/btag752 (PMC13722280; doi:10.1093/bioinformatics/btag752)
Supplement: Supplementary file 1 [file btag752_supplementary_data.pdf]

LoGicAl: Local ancestry and genotype calling uncertainty-aware ancestry-specific allele frequency estimation from admixed samples SUPPLEMENTARY MATERIAL

Jiongming Wang[1,2] and Sebastian Zöllner[1,2,3*]

[1]Department of Biostatistics, University of Michigan, Ann Arbor, MI 48109, USA.
[2]Center for Statistical Genetics, University of Michigan, Ann Arbor, MI 48109, USA.
[3]Department of Psychiatry, University of Michigan, Ann Arbor, MI 48109, USA.

*Corresponding author(s). E-mail(s): szoellne@umich.edu;

1 Supplementary figures

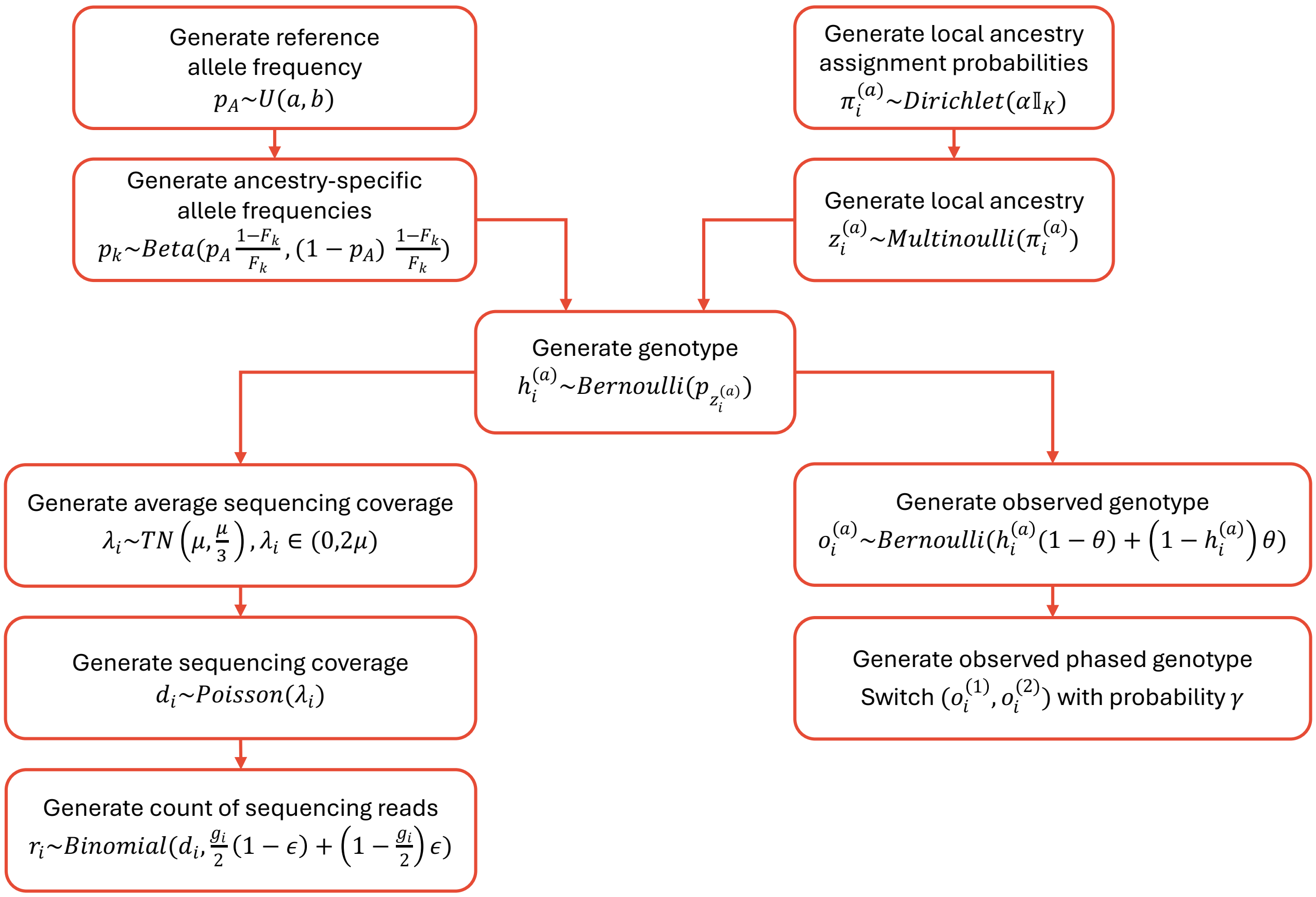

Figure S1: Simulation overview.

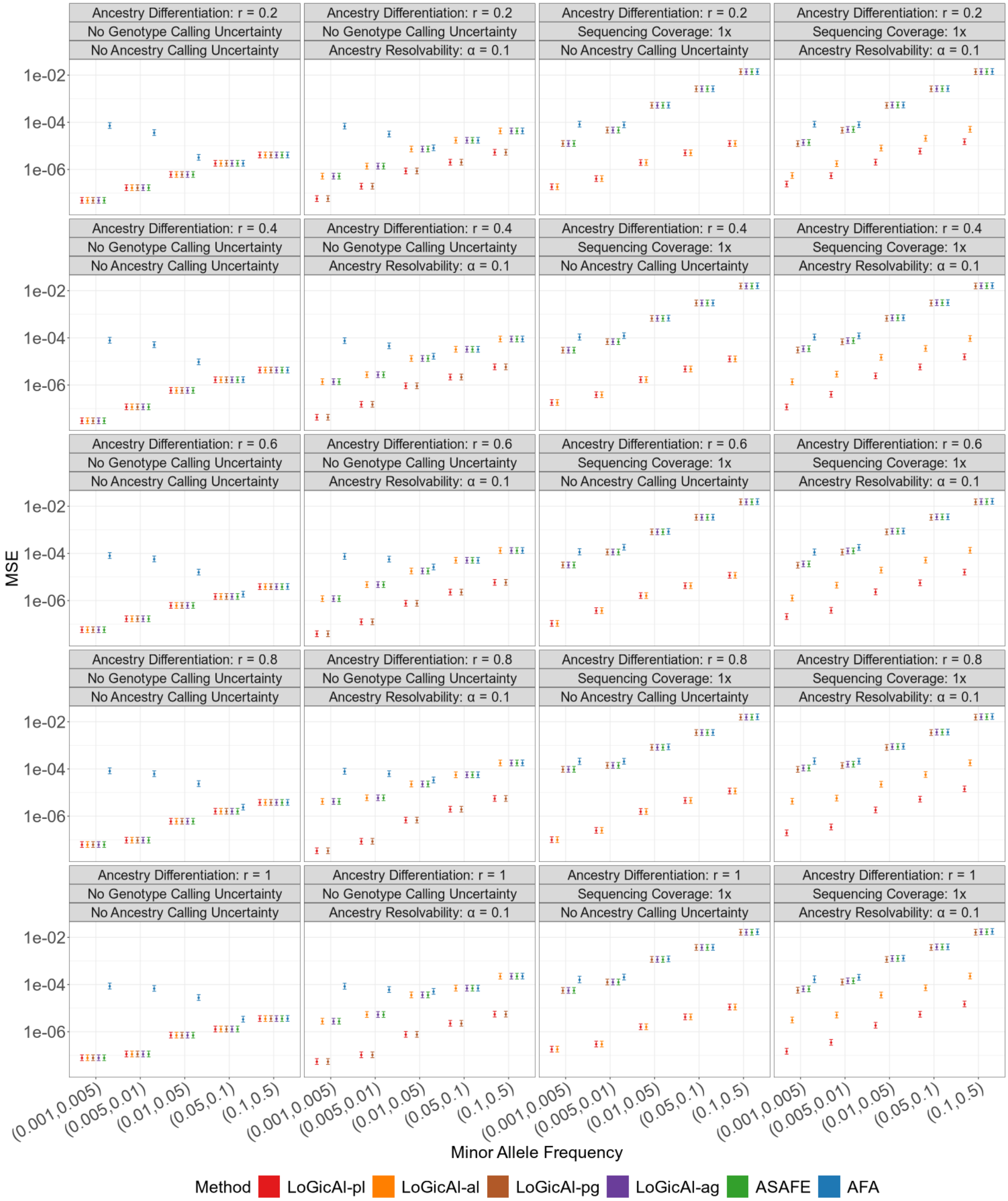

Figure S2: Mean squared error of ancestry-specific allele frequency estimation methods across uncertainty scenarios, allele frequency bins and levels of ancestral population differentiation. We simulated 100,000 unphased individuals from a three-way admixture model with intra-continental differentiation ($r = 0.2$) to inter-continental differentiation ($r = 1$). Methods compared include LoGicAl (LoGicAl-pl, LoGicAl-al, LoGicAl-pg, LoGicAl-ag), ASAFE, and AFA under four settings: no calling uncertainty, ancestry calling uncertainty only, genotype calling uncertainty only, and both ancestry and genotype calling uncertainty. Points denote replicate-averaged MSEs, and intervals indicate 95% confidence intervals for MSEs based on chi-squared approximation.

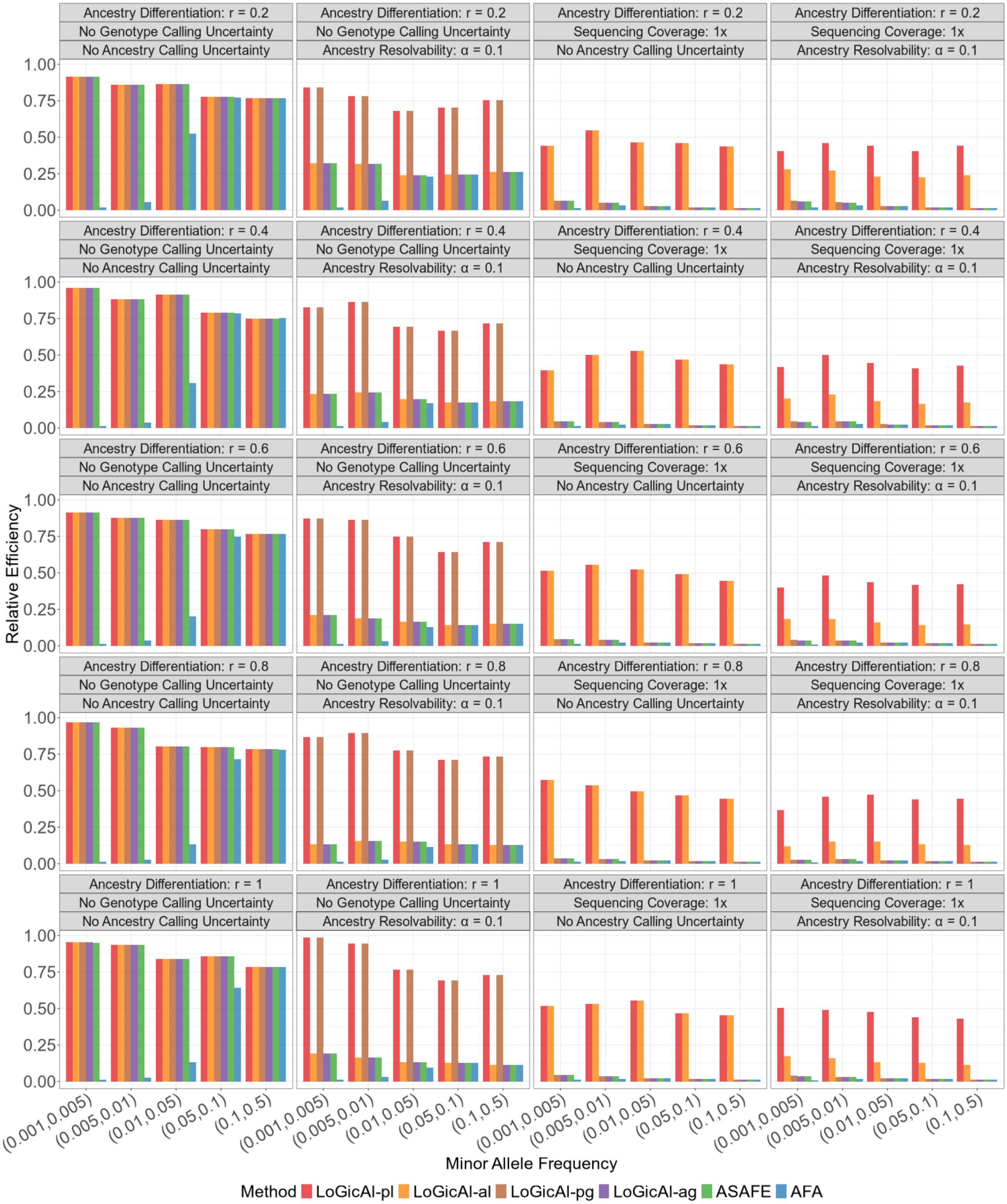

Figure S3: Relative efficiency of ancestry-specific allele frequency estimation methods across uncertainty scenarios, allele frequency bins and levels of ancestral population differentiation. We simulated 100,000 unphased individuals from a three-way admixture model with intra-continental differentiation ($r = 0.2$) to inter-continental differentiation ($r = 1$). Methods compared include LoGicAl (LoGicAl-pl, LoGicAl-al, LoGicAl-pg, LoGicAl-ag), ASAFE, and AFA under four settings: no calling uncertainty, ancestry calling uncertainty only, genotype calling uncertainty only, and both ancestry and genotype calling uncertainty. Bars denote relative efficiencies relative to the optimal estimator.

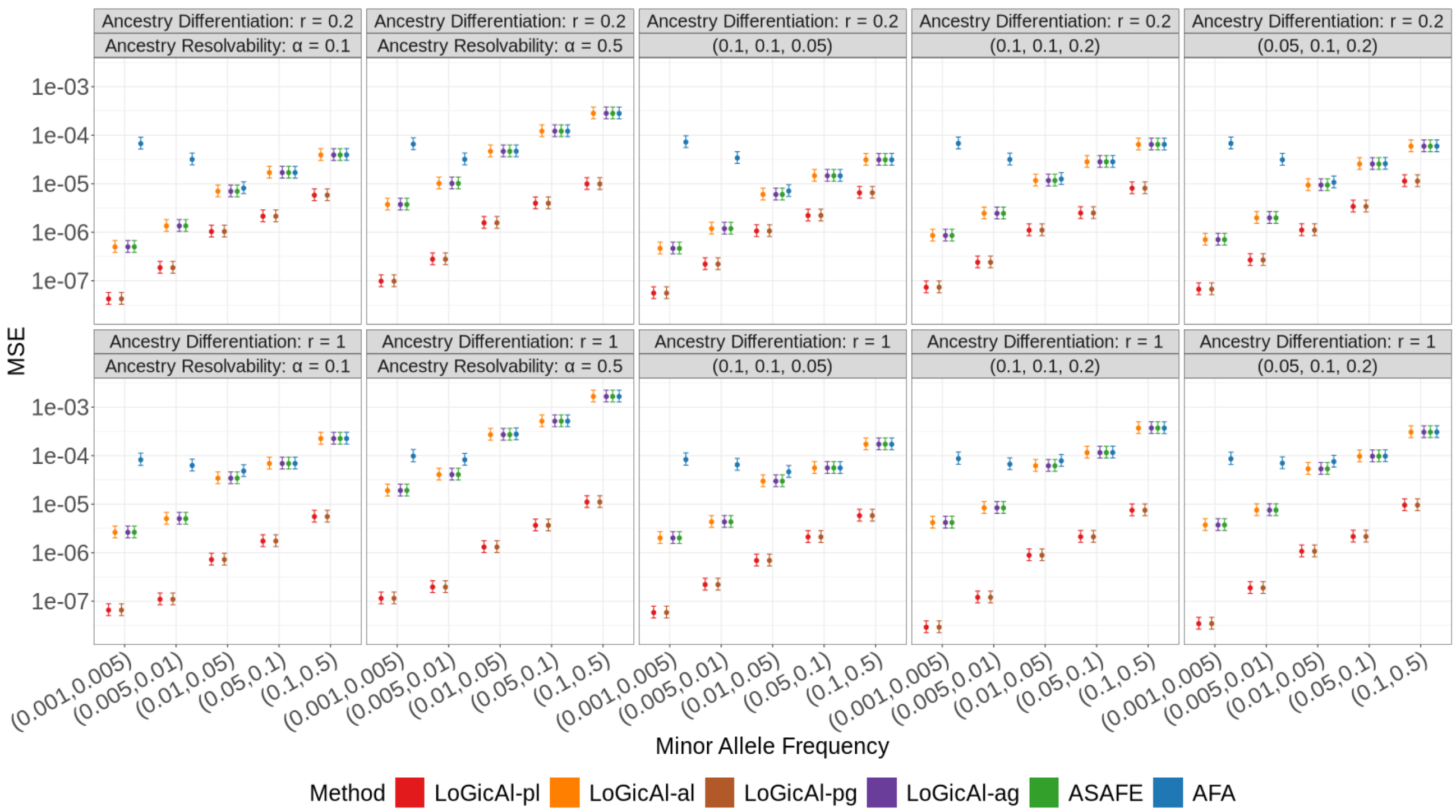

Figure S4: Mean squared error of ancestry-specific allele frequency estimation methods across ancestry calling uncertainty scenarios, allele frequency bins and levels of ancestral population differentiation. We simulated 100,000 unphased individuals from a three-way admixture model with intra-continental differentiation ($r = 0.2$) and inter-continental differentiation ($r = 1$) while assuming no genotype calling uncertainty. Methods compared include LoGicAl (LoGicAl-pl, LoGicAl-al, LoGicAl-pg, LoGicAl-ag), ASAFE, and AFA under five ancestry calling uncertainty settings, with two symmetric Dirichlet scenarios with $\alpha = 0.1$ and $\alpha = 0.5$ and three asymmetric scenarios with Dirichlet parameters $(0.1, 0.1, 0.05)$, $(0.1, 0.1, 0.2)$, and $(0.05, 0.1, 0.2)$. Points denote replicate-averaged MSEs, and intervals indicate 95% confidence intervals for MSEs based on chi-squared approximation.

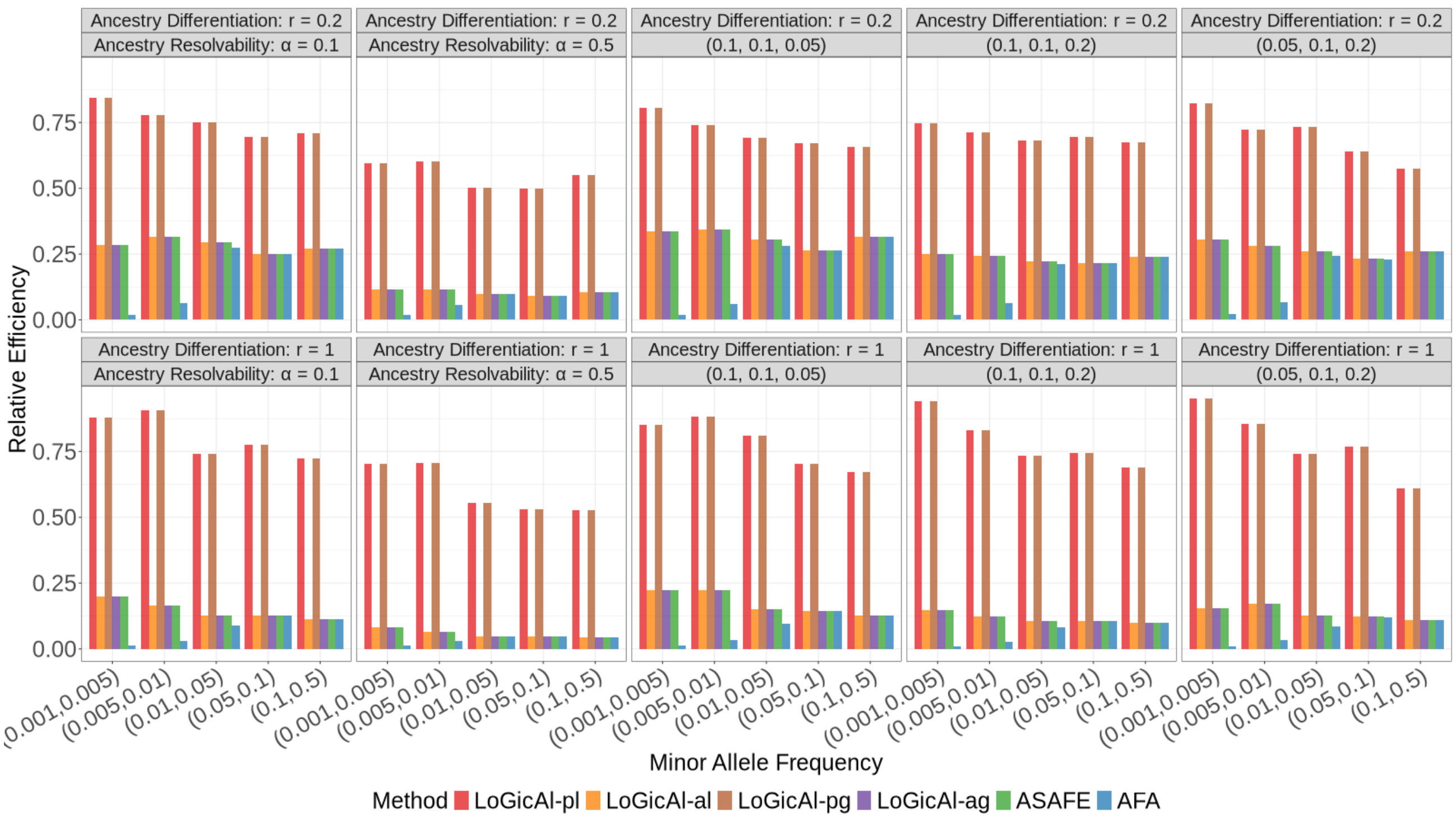

Figure S5: Relative efficiency of ancestry-specific allele frequency estimation methods across ancestry calling uncertainty scenarios, allele frequency bins and levels of ancestral population differentiation. We simulated 100,000 unphased individuals from a three-way admixture model with intra-continental differentiation ($r = 0.2$) and inter-continental differentiation ($r = 1$) while assuming no genotype calling uncertainty. Methods compared include LoGicAl (LoGicAl-pl, LoGicAl-al, LoGicAl-pg, LoGicAl-ag), ASAFE, and AFA under five ancestry calling uncertainty settings, with two symmetric Dirichlet scenarios with $\alpha = 0.1$ and $\alpha = 0.5$ and three asymmetric scenarios with Dirichlet parameters $(0.1, 0.1, 0.05)$, $(0.1, 0.1, 0.2)$, and $(0.05, 0.1, 0.2)$. Bars denote relative efficiencies relative to the optimal estimator.

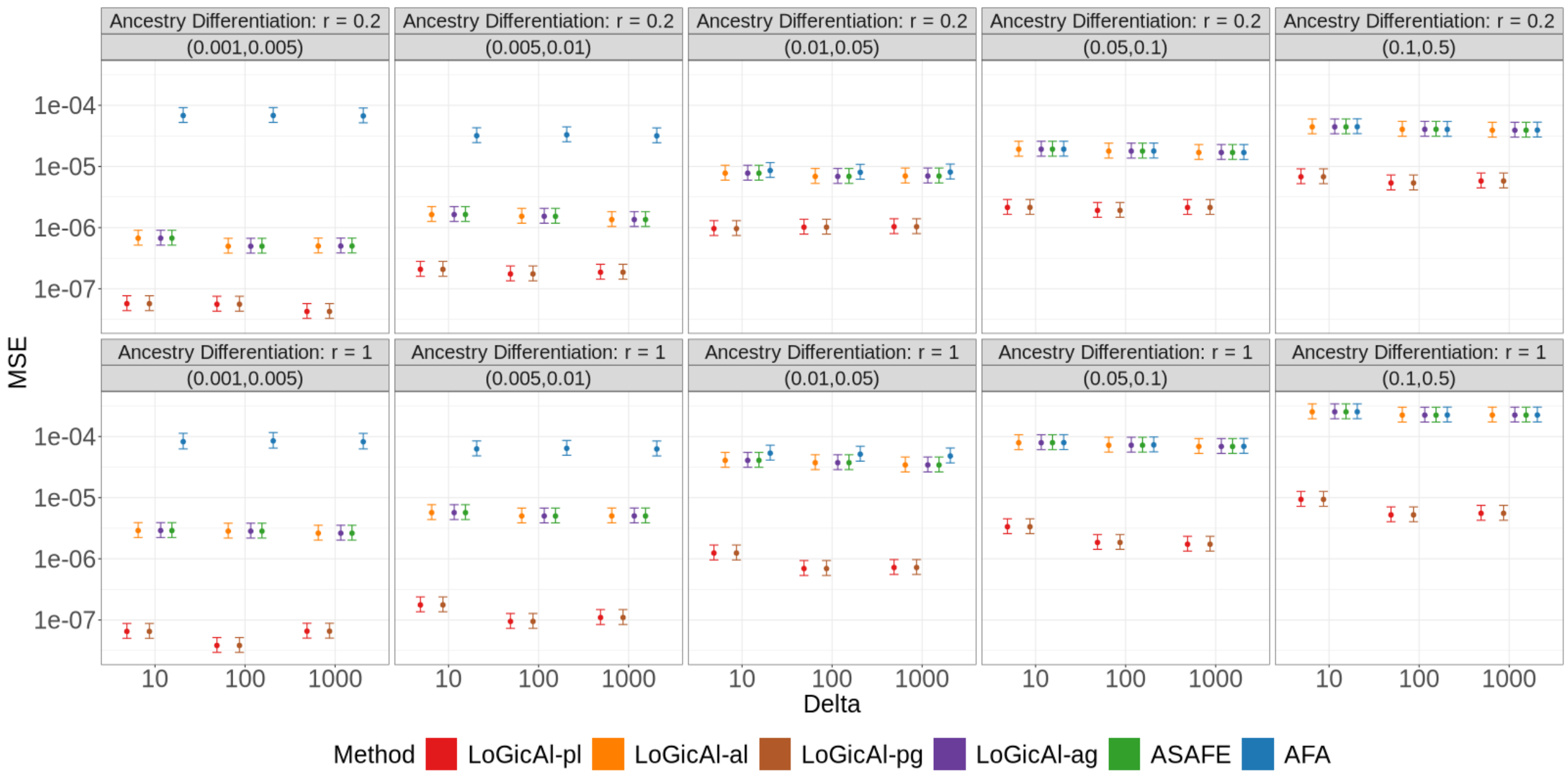

Figure S6: Mean squared error of ancestry-specific allele frequency estimation methods across quality of estimated local ancestry assignment probabilities, allele frequency bins and levels of ancestral population differentiation. We simulated 100,000 unphased individuals from a three-way admixture model with intra-continental differentiation ($r = 0.2$) and inter-continental differentiation ($r = 1$) while assuming ancestry calling uncertainty only. Methods compared include LoGicAl (LoGicAl-pl, LoGicAl-al, LoGicAl-pg, LoGicAl-ag), ASAFE, and AFA. Points denote replicate-averaged MSEs, and intervals indicate 95% confidence intervals for MSEs based on chi-squared approximation.

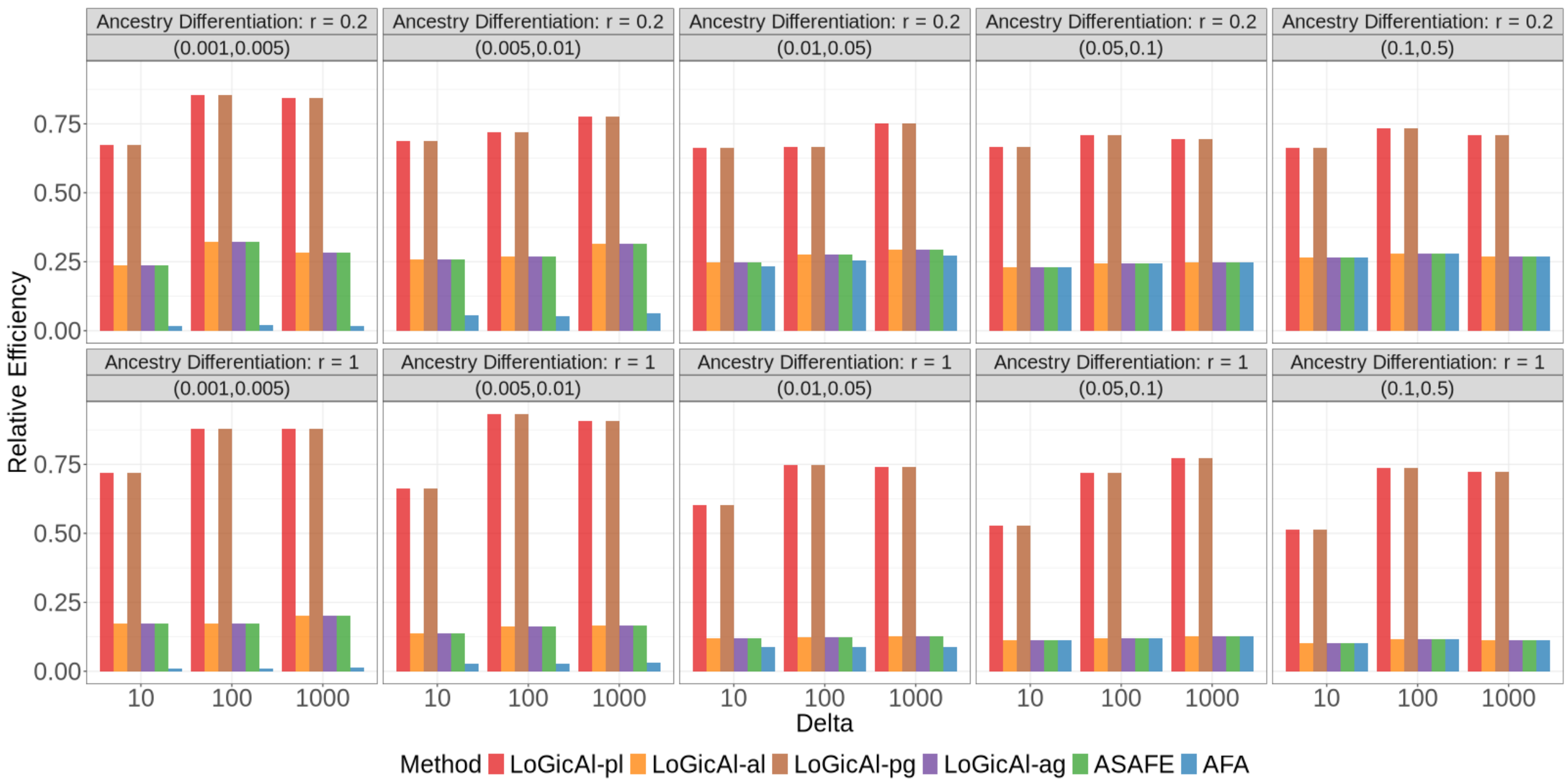

Figure S7: Relative efficiency of ancestry-specific allele frequency estimation methods across quality of estimated local ancestry assignment probabilities, allele frequency bins and levels of ancestral population differentiation. We simulated 100, 000 unphased individuals from a three-way admixture model with intra-continental differentiation ($r = 0.2$) and inter-continental differentiation ($r = 1$) while assuming ancestry calling uncertainty only. Methods compared include LoGicAl (LoGicAl-pl, LoGicAl-al, LoGicAl-pg, LoGicAl-ag), ASAFE, and AFA. Bars denote relative efficiencies relative to the optimal estimator.

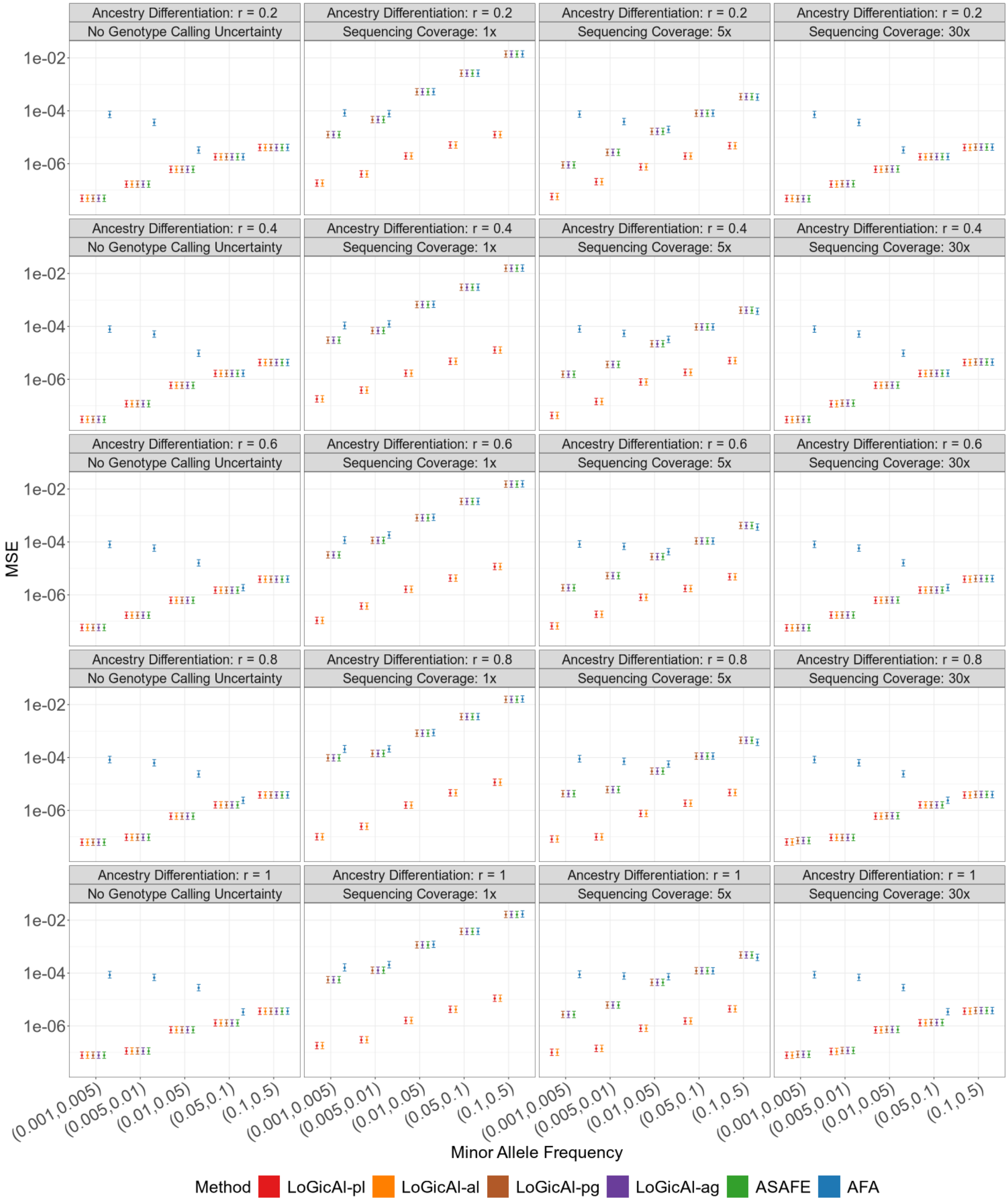

Figure S8: Mean squared error of ancestry-specific allele frequency estimation methods across sequencing depths, allele frequency bins and levels of ancestral population differentiation. We simulated 100,000 unphased individuals from a three-way admixture model with intra-continental differentiation ($r = 0.2$) to inter-continental differentiation ($r = 1$) while assuming no ancestry inference uncertainty. Methods compared include LoGicAl (LoGicAl-pl, LoGicAl-al, LoGicAl-pg, LoGicAl-ag), ASAFE, and AFA. Points denote replicate-averaged MSEs, and intervals indicate 95% confidence intervals for MSEs based on chi-squared approximation.

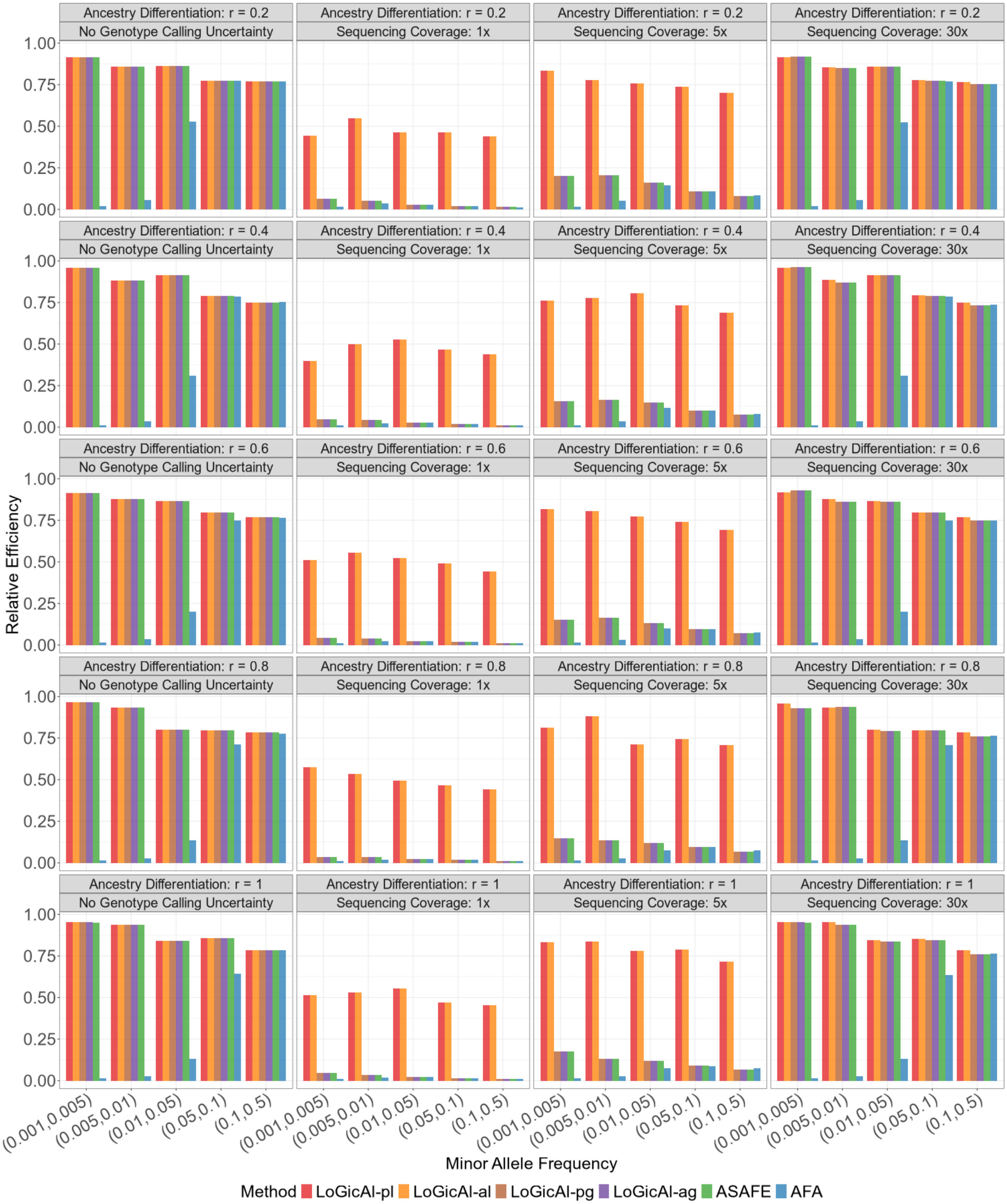

Figure S9: Relative efficiency of ancestry-specific allele frequency estimation methods across sequencing depths, allele frequency bins and ancestral population differentiation. We simulated 100,000 unphased individuals from a three-way admixture model with intra-continental differentiation ($r = 0.2$) to inter-continental differentiation ($r = 1$) while assuming no ancestry inference uncertainty. Methods compared include LoGicAl (LoGicAl-pl, LoGicAl-al, LoGicAl-pg, LoGicAl-ag), ASAFE, and AFA. Bars denote relative efficiencies relative to the optimal estimator.

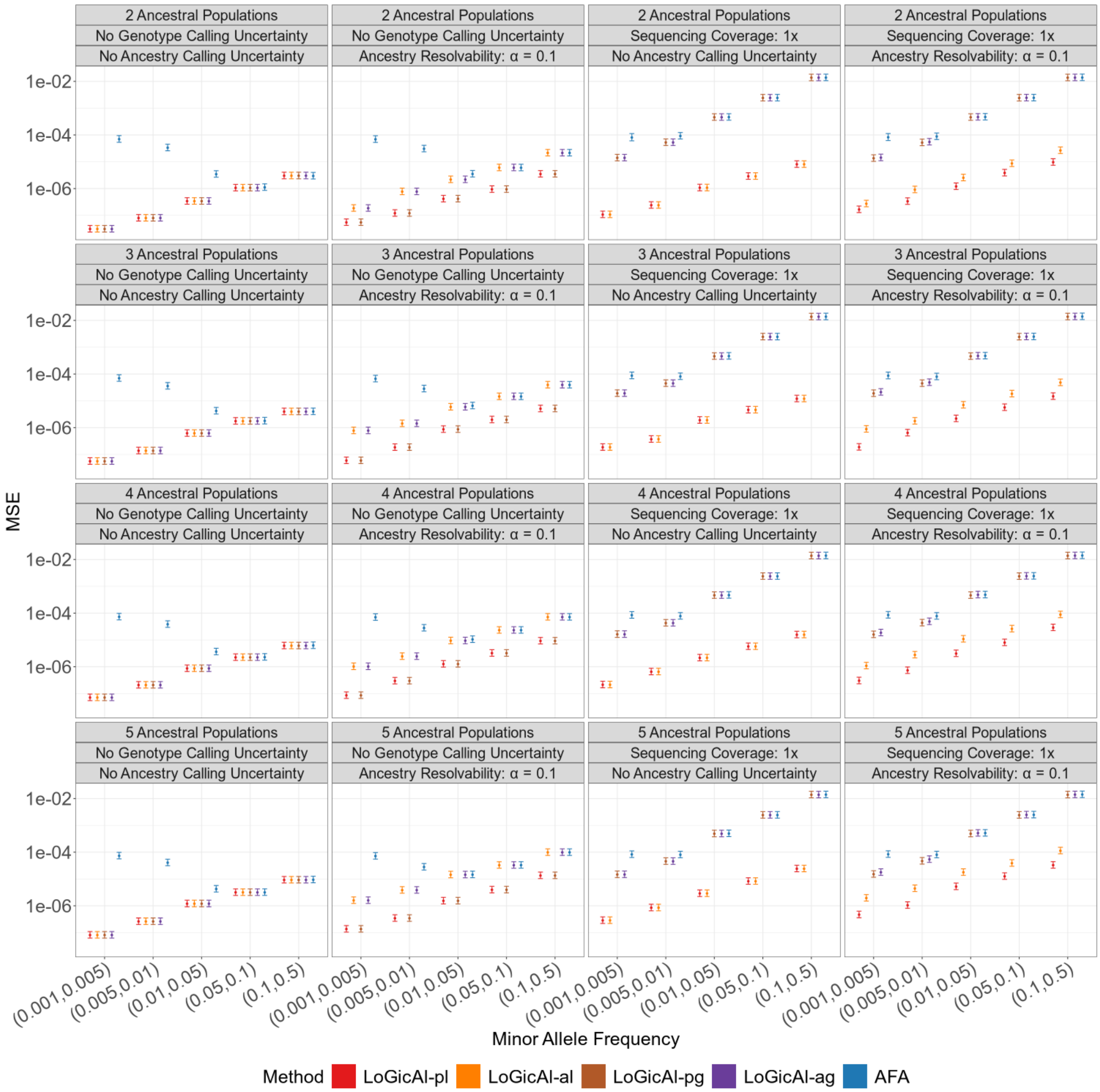

Figure S10: Mean squared error of ancestry-specific allele frequency estimation methods across uncertainty scenarios, allele frequency bins and numbers of ancestral populations. We simulated 100, 000 unphased individuals under two- to five-way admixture with intra-continental differentiation. Methods compared include LoGicAl (LoGicAl-pl, LoGicAl-al, LoGicAl-pg, LoGicAl-ag) and AFA under four settings: no calling uncertainty, ancestry calling uncertainty only, genotype calling uncertainty only, and both ancestry and genotype calling uncertainty. ASAFE is not included as it is developed for three-way (and two-way) admixture scenario only. Points denote replicate-averaged MSEs, and intervals indicate 95% confidence intervals for MSEs based on chi-squared approximation.

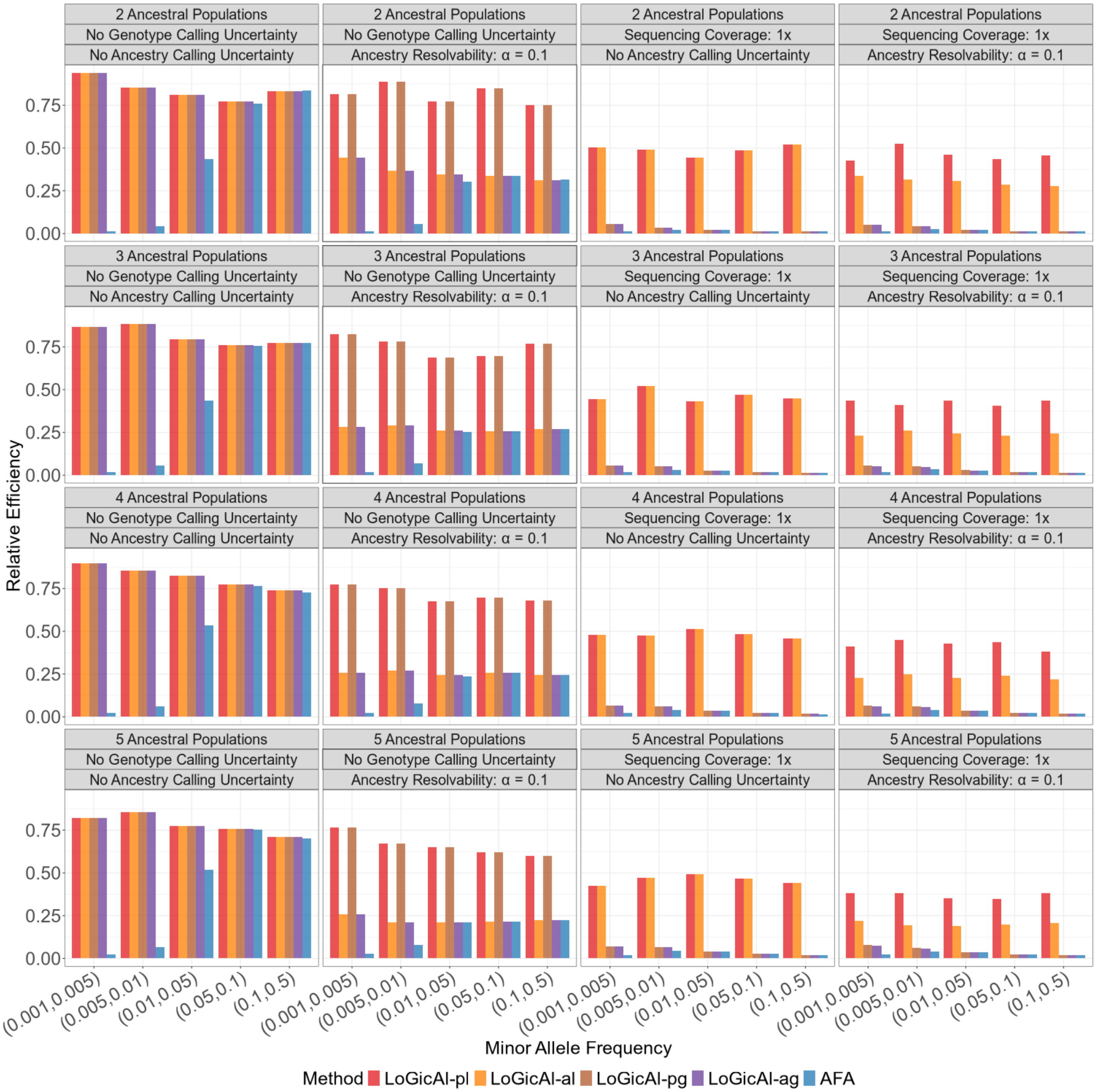

Figure S11: Relative efficiency of ancestry-specific allele frequency estimation methods across uncertainty scenarios, allele frequency bins and numbers of ancestral populations. We simulated 100,000 unphased individuals under two- to five-way admixture with intra-continental differentiation. Methods compared include LoGicAl (LoGicAl-pl, LoGicAl-al, LoGicAl-pg, LoGicAl-ag) and AFA under four settings: no calling uncertainty, ancestry calling uncertainty only, genotype calling uncertainty only, and both ancestry and genotype calling uncertainty. ASAFE is not included as it is developed for three-way (and two-way) admixture scenario only. Bars denote relative efficiencies relative to the optimal estimator.

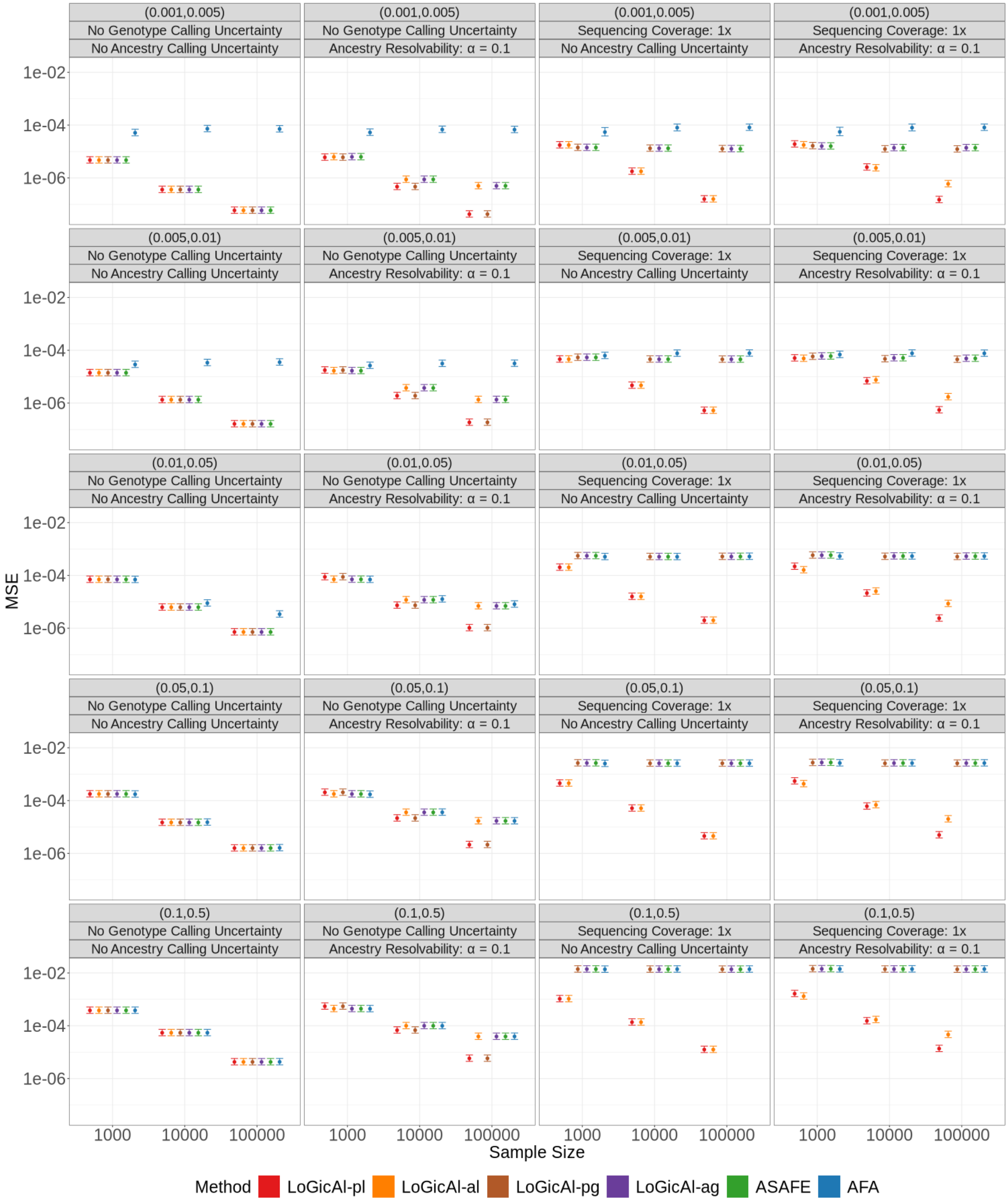

Figure S12: Mean squared error of ancestry-specific allele frequency estimation methods across sample sizes, uncertainty scenarios and allele frequency bins. We simulated 1,000, 10,000, and 100,000 unphased individuals from a three-way admixture model with intra-continental differentiation ($r = 0.2$). Methods compared include LoGicAl (LoGicAl-pl, LoGicAl-al, LoGicAl-pg, LoGicAl-ag), ASAFE, and AFA under four settings: no calling uncertainty, ancestry calling uncertainty only, genotype calling uncertainty only, and both ancestry and genotype calling uncertainty. Points denote replicate-averaged MSEs, and intervals indicate 95% confidence intervals for MSEs based on chi-squared approximation.

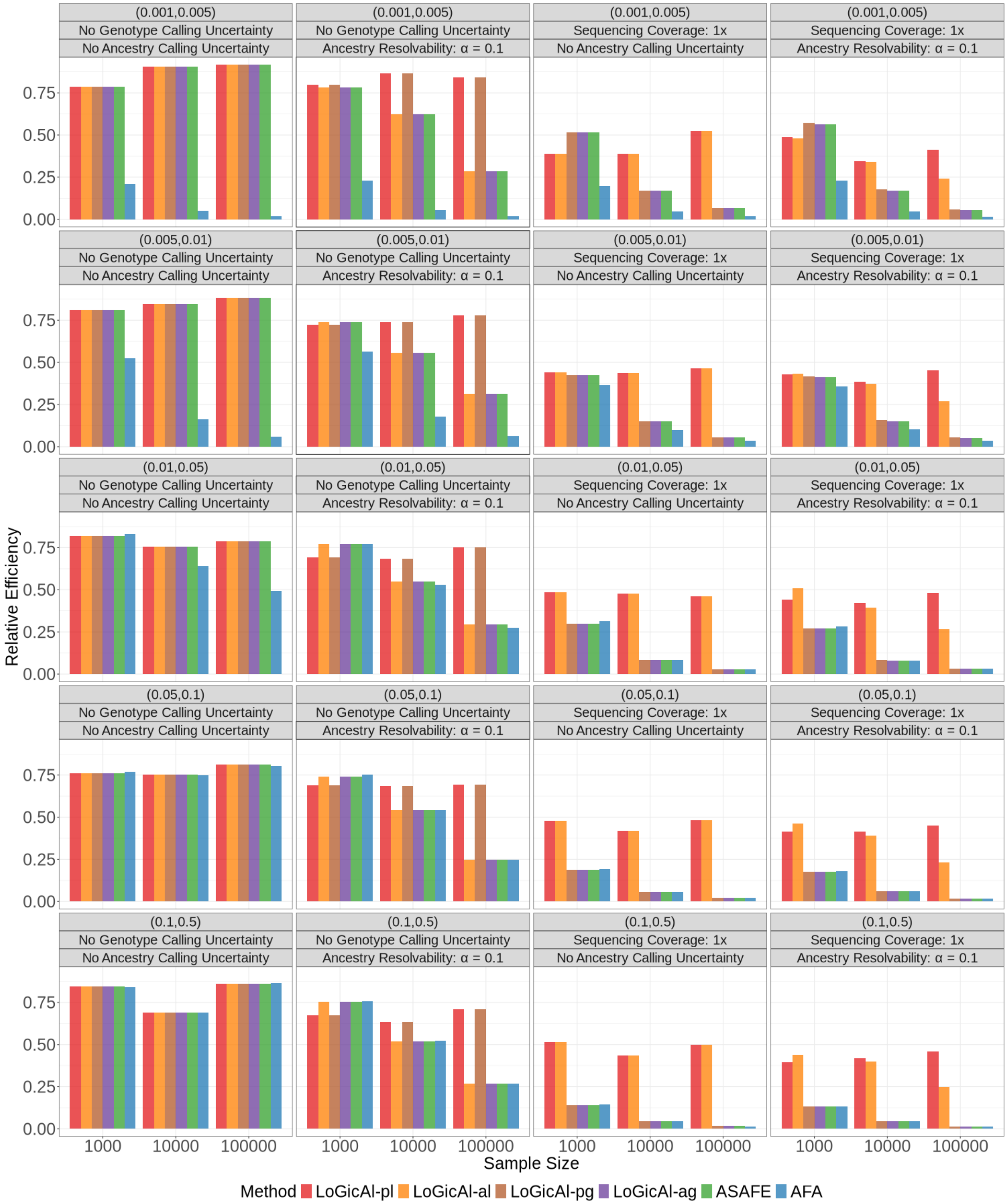

Figure S13: Relative efficiency of ancestry-specific allele frequency estimation methods across sample sizes, uncertainty scenarios and allele frequency bins. We simulated 1,000, 10,000, and 100,000 unphased individuals from a three-way admixture model with intra-continental differentiation ($r = 0.2$). Methods compared include LoGicAl (LoGicAl-pl, LoGicAl-al, LoGicAl-pg, LoGicAl-ag), ASAFE, and AFA under four settings: no calling uncertainty, ancestry calling uncertainty only, genotype calling uncertainty only, and both ancestry and genotype calling uncertainty. Bars denote relative efficiencies relative to the optimal estimator.

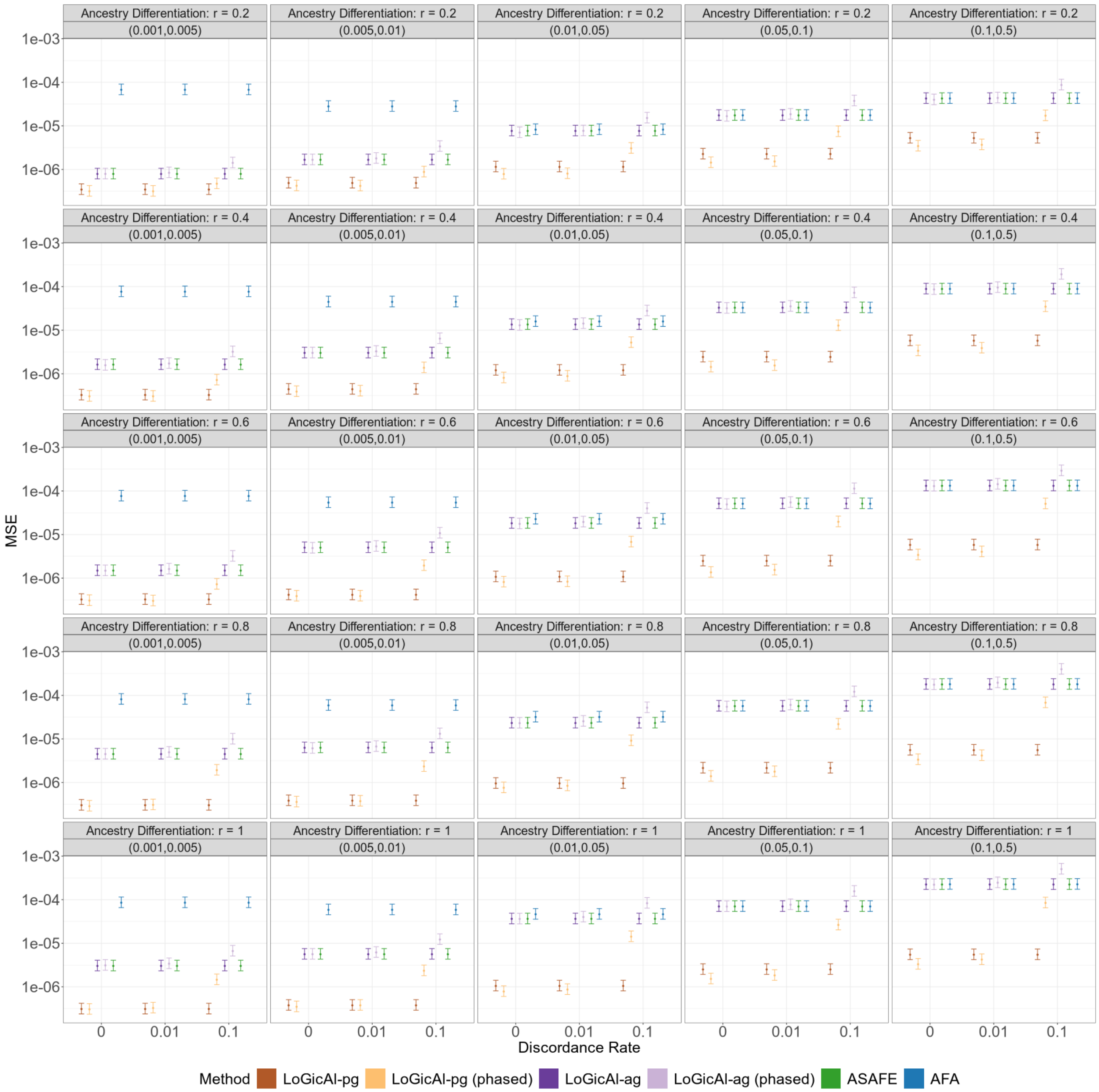

Figure S14: Mean squared error of ancestry-specific allele frequency estimation methods across discordance rates, allele frequency bins and levels of ancestral population differentiation. We simulated $100,000$ phased individuals from a three-way admixture model under a scenario with probabilistic ancestry calling ($\alpha = 0.1$) and a realistic genotyping error rate ($\theta = 5 \times 10^{-4}$). Methods compared include LoGicAl (LoGicAl-pg, LoGicAl-pg (phased), LoGicAl-ag, LoGicAl-ag (phased)), ASAFE and AFA. Points denote replicate-averaged MSEs, and intervals indicate 95% confidence intervals for MSEs based on chi-squared approximation.

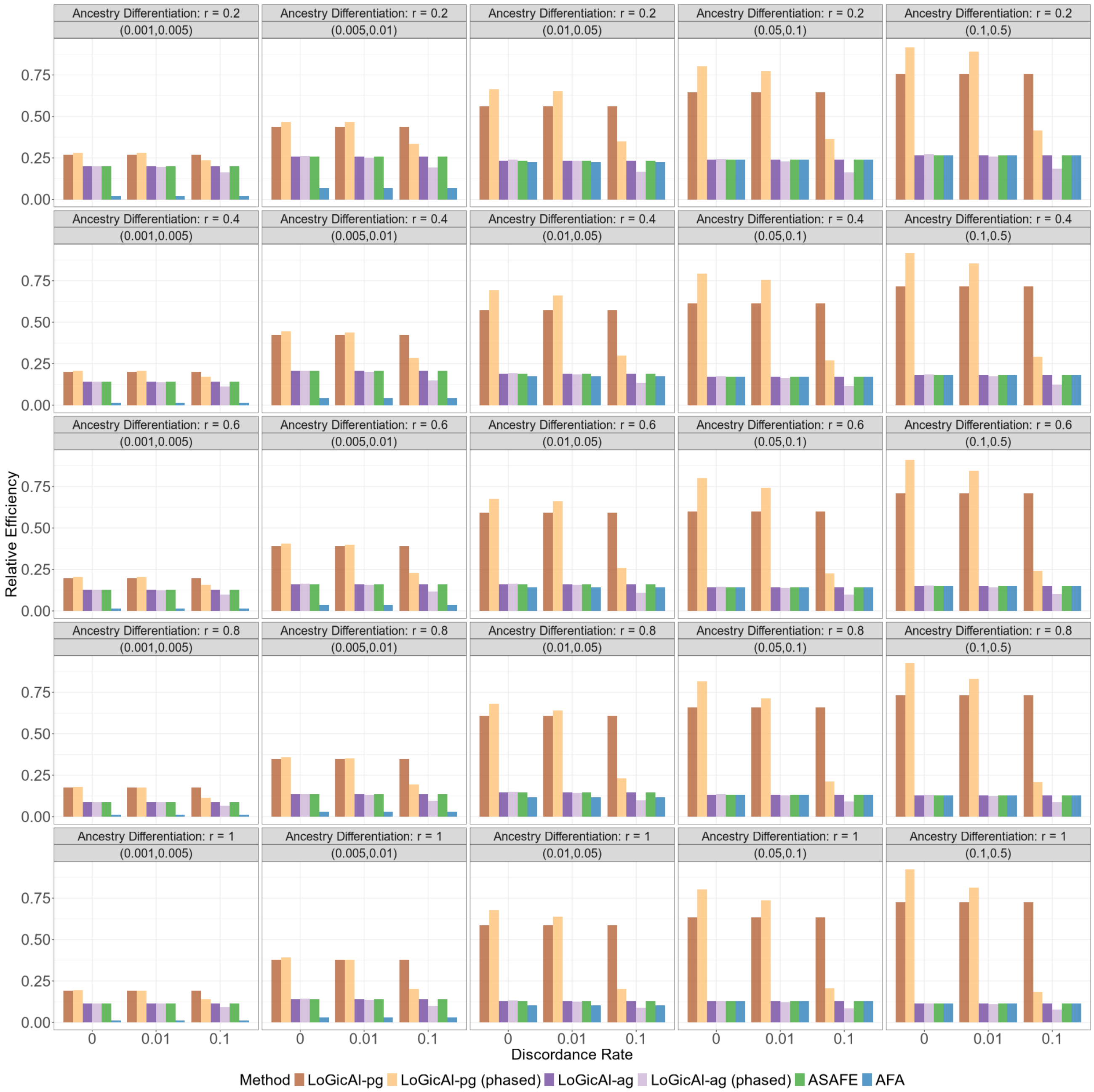

Figure S15: Relative efficiency of ancestry-specific allele frequency estimation methods across discordance rates, allele frequency bins and levels of ancestral population differentiation. We simulated $100,000$ phased individuals from a three-way admixture model under a scenario with probabilistic ancestry calling ($\alpha = 0.1$) and a realistic genotyping error rate ($\theta = 5 \times 10^{-4}$). Methods compared include LoGicAl (LoGicAl-pg, LoGicAl-pg (phased), LoGicAl-ag, LoGicAl-ag (phased)), ASAFE and AFA. Bars denote relative efficiencies relative to the optimal estimator.

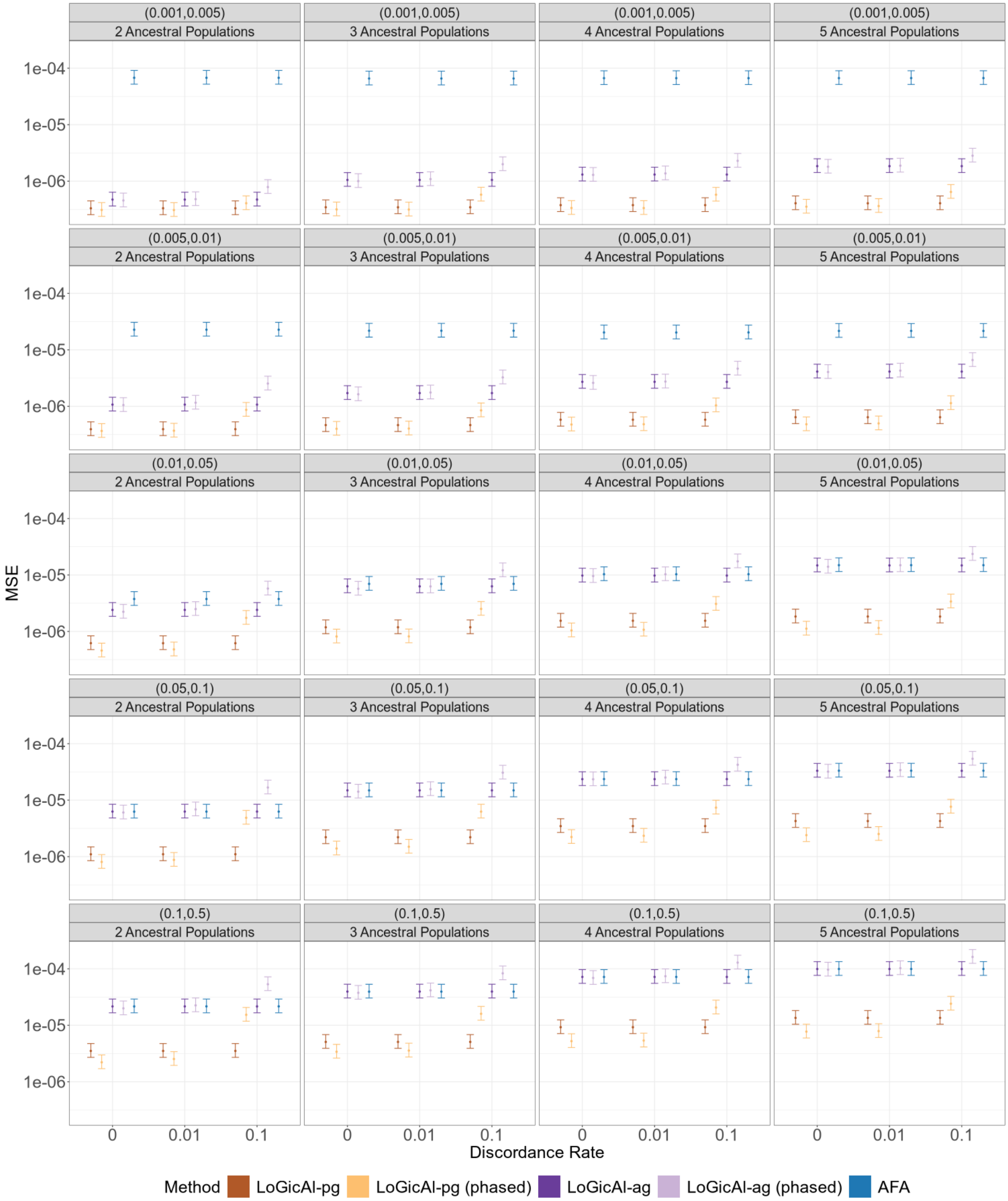

Figure S16: Mean squared error of ancestry-specific allele frequency estimation methods across discordance rates, allele frequency bins and numbers of ancestral populations. We simulated 100,000 phased individuals under two- to five-way admixture with intra-continental differentiation. Methods compared include LoGicAl (LoGicAl-pg, LoGicAl-pg (phased), LoGicAl-ag, LoGicAl-ag (phased)) and AFA under a scenario with probabilistic ancestry calling ($\alpha = 0.1$) and a realistic genotyping error rate ($\theta = 5 \times 10^{-4}$). ASAFE is not included as it is developed for three-way (and two-way) admixture scenario only. Points denote replicate-averaged MSEs, and intervals indicate 95% confidence intervals for MSEs based on chi-squared approximation.

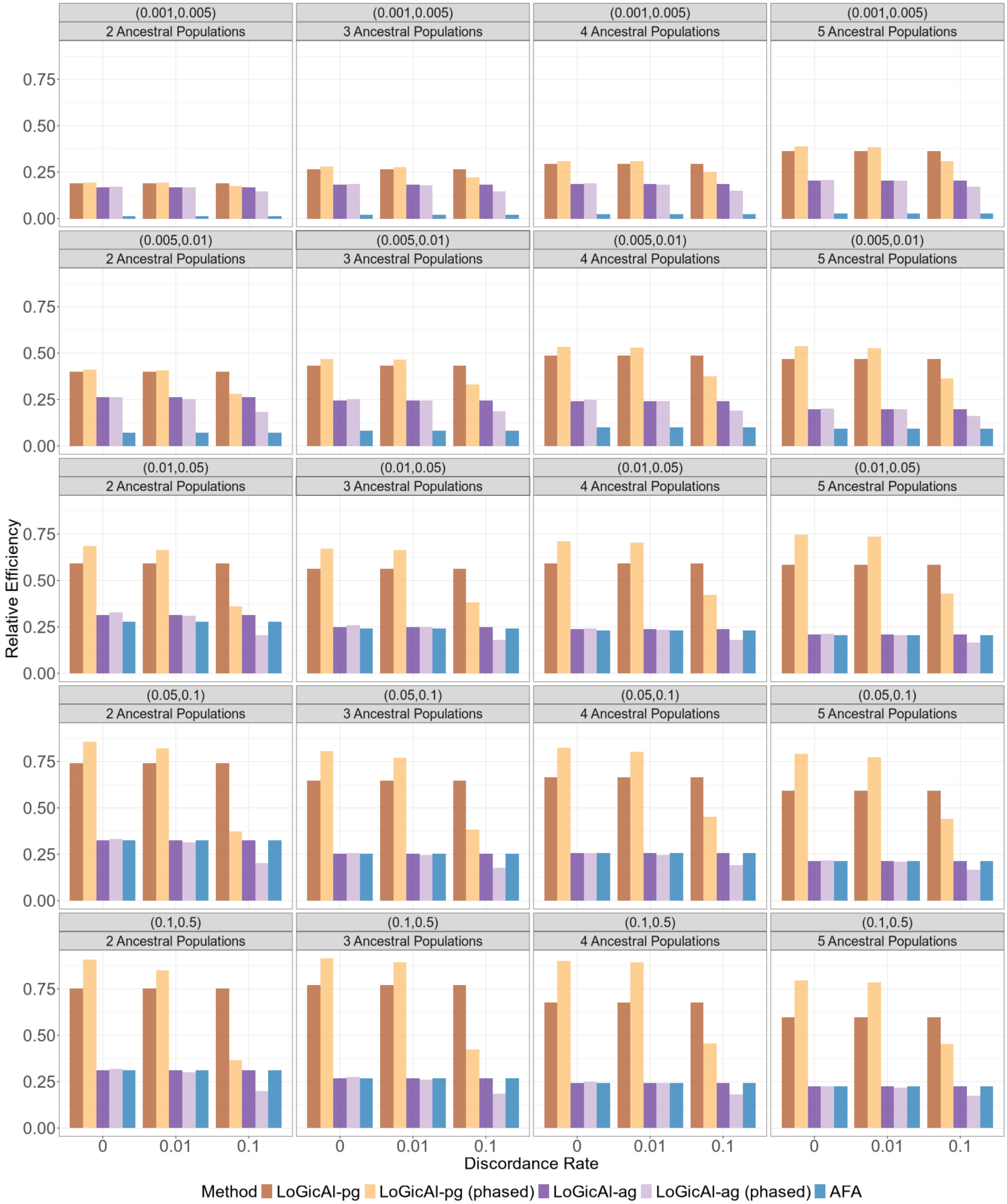

Figure S17: Relative efficiency of ancestry-specific allele frequency estimation methods across discordance rates, allele frequency bins and numbers of ancestral populations. We simulated $100,000$ phased individuals under two- to five-way admixture with intra-continental differentiation. Methods compared include LoGicAl (LoGicAl-pg, LoGicAl-pg (phased), LoGicAl-ag, LoGicAl-ag (phased)) and AFA under a scenario with probabilistic ancestry calling ($\alpha = 0.1$) and a realistic genotyping error rate ($\theta = 5 \times 10^{-4}$). ASAFE is not included as it is developed for three-way (and two-way) admixture scenario only. Bars denote relative efficiencies relative to the optimal estimator.

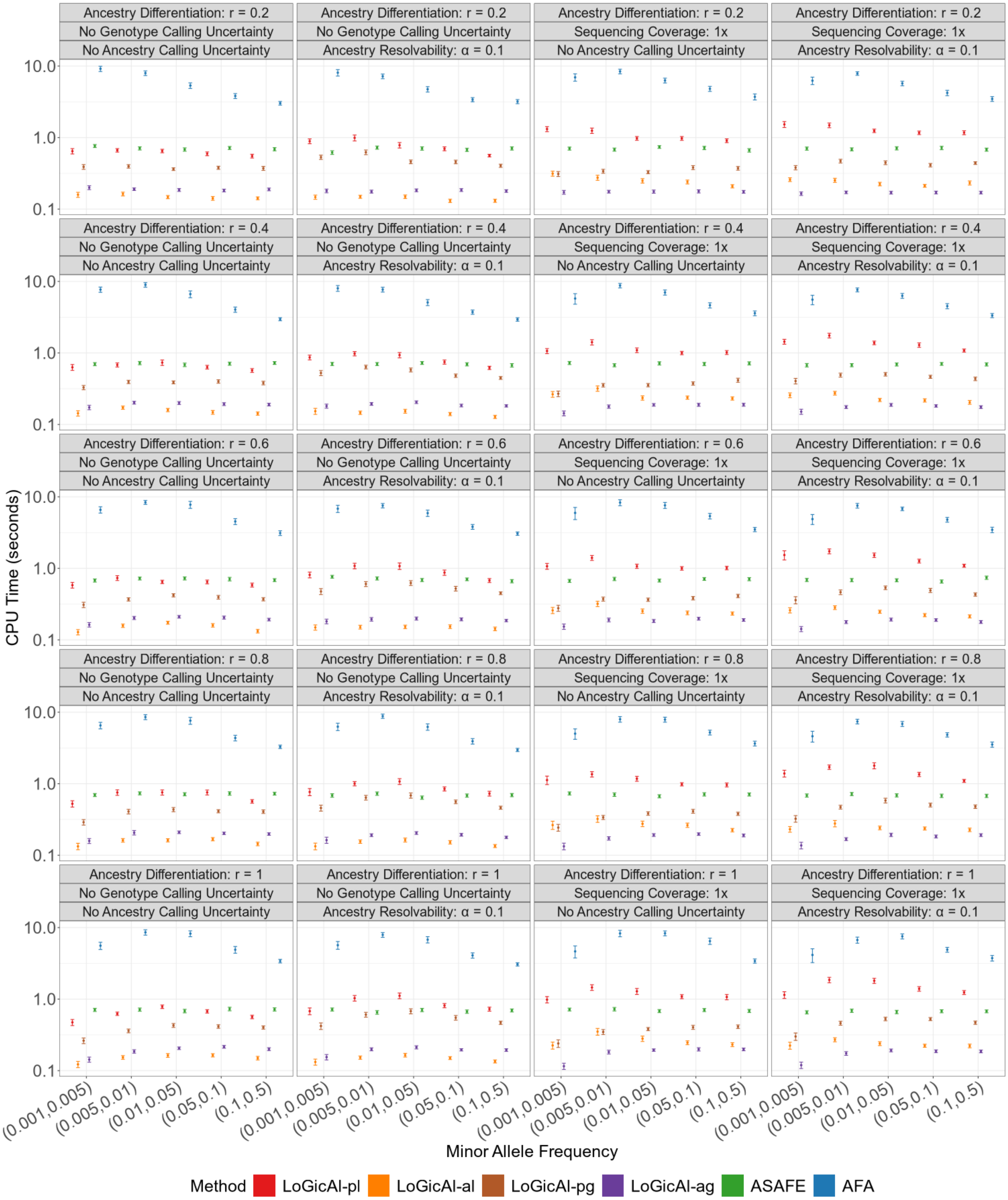

Figure S18: CPU runtime (seconds) of ancestry-specific allele frequency estimation methods across uncertainty scenarios, allele frequency bins and levels of ancestral population differentiation. We simulated 100,000 unphased individuals from a three-way admixture model with intra-continental differentiation ($r = 0.2$) to inter-continental differentiation ($r = 1$). Methods compared include LoGicAl (LoGicAl-pl, LoGicAl-al, LoGicAl-pg, LoGicAl-ag), ASAFE, and AFA under four settings: no calling uncertainty, ancestry calling uncertainty only, genotype calling uncertainty only, and both ancestry and genotype calling uncertainty. Points denote replicate-averaged runtime, and intervals indicate 95% confidence intervals for runtime based on normal approximation.

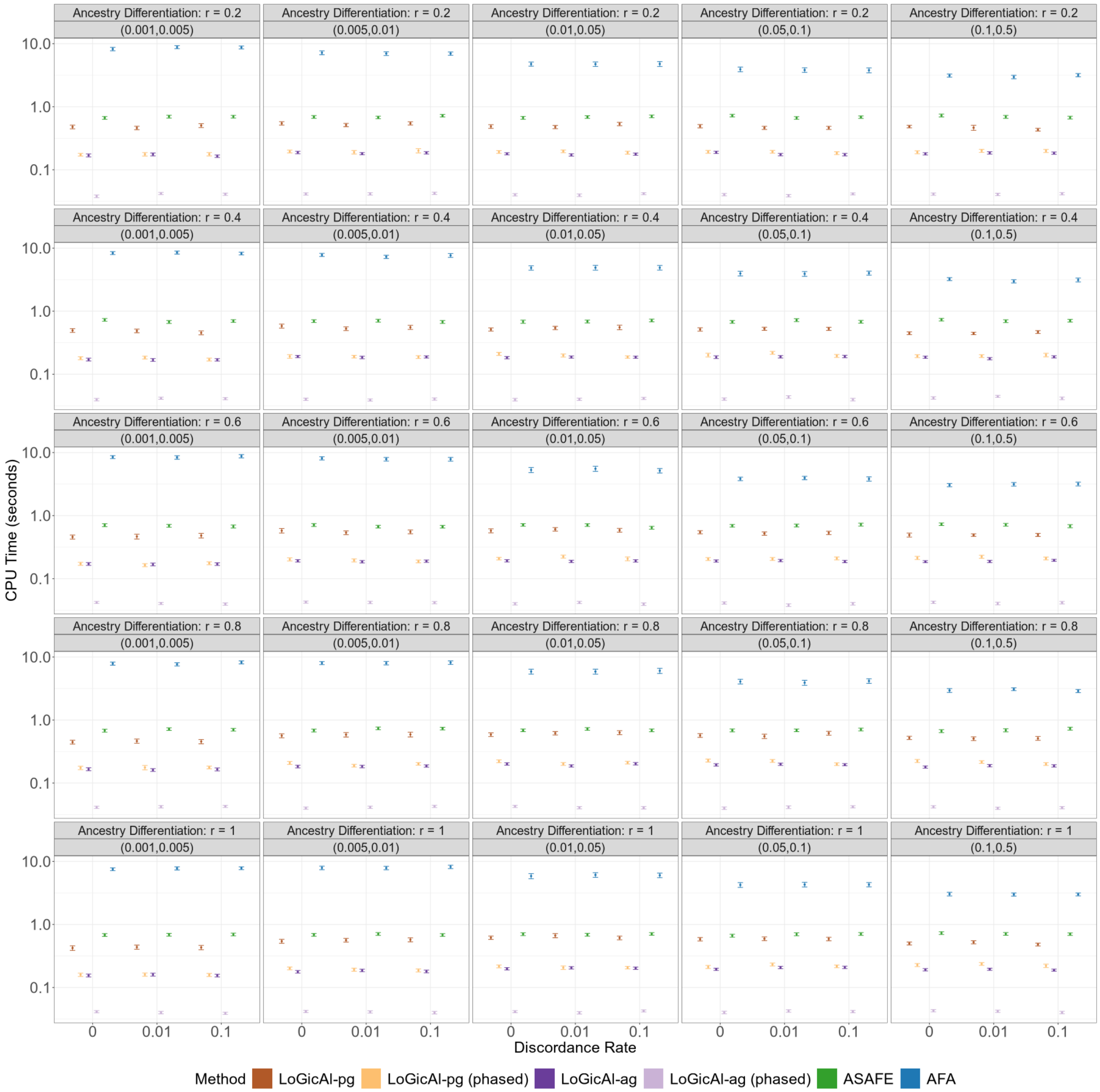

Figure S19: CPU runtime (seconds) of ancestry-specific allele frequency estimation methods across discordance rates, allele frequency bins and levels of ancestral population differentiation. We simulated 100, 000 phased individuals from a three-way admixture model under a scenario with probabilistic ancestry calling ($\alpha = 0.1$) and a realistic genotyping error rate ($\theta = 5 \times 10^{-4}$). Methods compared include LoGicAl (LoGicAl-pg, LoGicAl-pg (phased), LoGicAl-ag, LoGicAl-ag (phased)), ASAFE and AFA. Points denote replicate-averaged runtime, and intervals indicate 95% confidence intervals for runtime based on normal approximation.

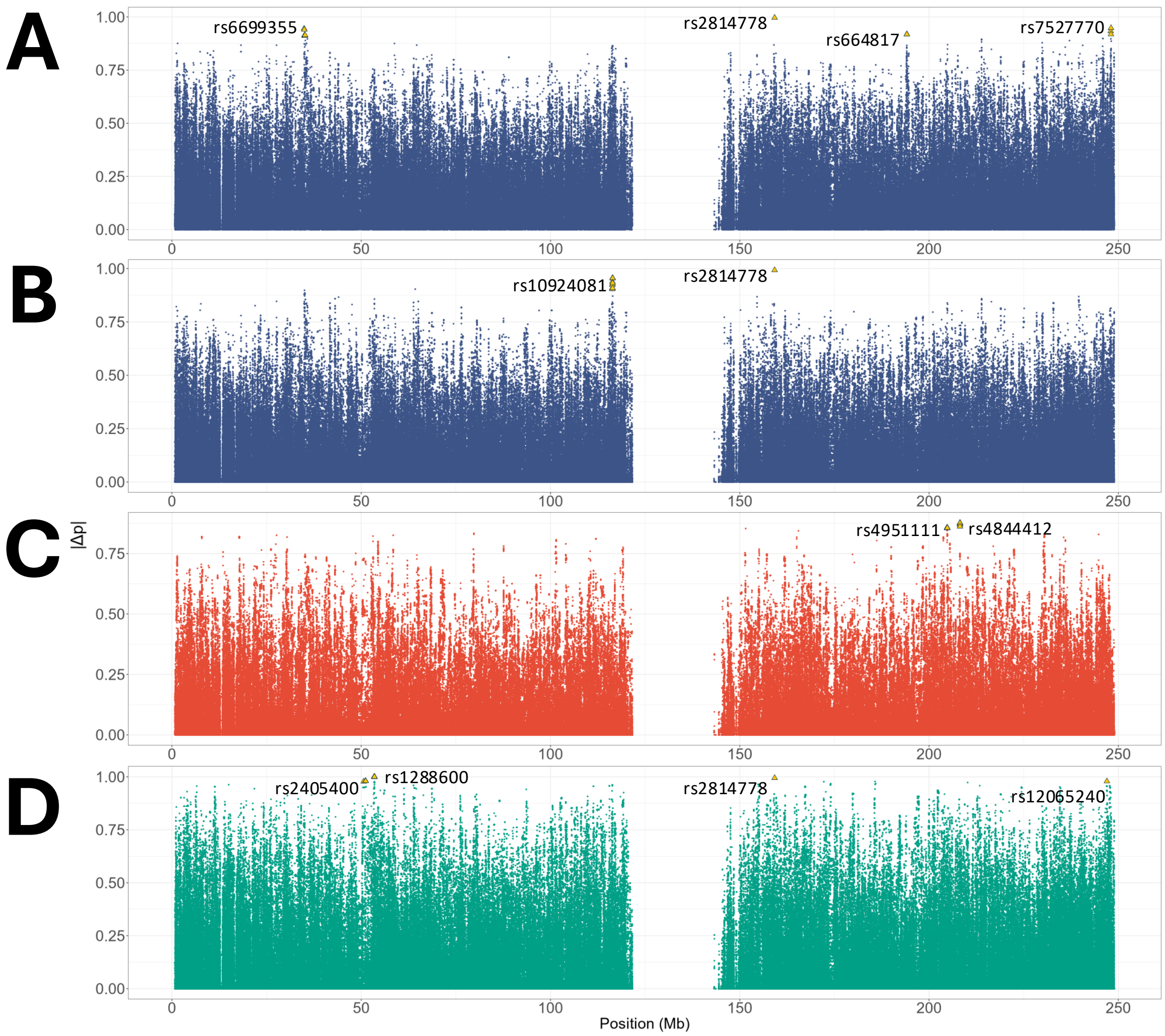

Figure S20: Absolute differences of ancestry-specific allele frequency estimates across chromosome 1. (A) Absolute differences between African and European ancestral estimates in 1kGP admixed African cohorts. (B) Absolute differences between African and European ancestral estimates in 1kGP admixed American cohorts. (C) Absolute differences between Native American and European ancestral estimates in 1kGP admixed American cohorts. (D) Absolute differences between Native American and African ancestral estimates in 1kGP admixed American cohorts. Outliers (yellow) denote the top 10 most differentiated variants per comparison; among these, the most differentiated variant within 1 Mb window is labeled by its rsID.

2 Supplementary tables

Table S1: Dirichlet regression estimates of resolvability of local ancestry assignment across 1000 Genomes Project individuals. We fitted an intercept-only Dirichlet regression model to the posterior probabilities of assigning each ancestral superpopulation generated by RFMix, using the `DirichReg()` function from the `DirichletReg` R package. We calculated the estimated α parameters as the exponential of the coefficients corresponding to each superpopulation from the fitted model.

Superpopulation	AFR	SAS	EAS	EUR	AMR	OCN	MES
$\hat{\alpha}$	0.118	0.107	0.110	0.118	0.094	0.092	0.092

AFR: Sub-Saharan Africa, SAS: Central & South Asia, EAS: East Asia, EUR: Europe, AMR: Native America, OCN: Oceania, MES: Middle East.

Table S2: CPU runtime (seconds) of ancestry-specific allele frequency estimation methods across allele frequency bins and uncertainty scenarios. We simulated $100,000$ unphased individuals from a three-way admixture model with inter-continental differentiation ($r = 1$). Methods compared include LoGicAl (LoGicAl-pl, LoGicAl-al, LoGicAl-pg, LoGicAl-ag), ASAFE, and AFA under four settings: (O) no calling uncertainty, (A) ancestry calling uncertainty only, (G) genotype calling uncertainty only, and (A+G) both ancestry and genotype calling uncertainty. Runtimes are reported for a single variant and are averaged over 100 simulation replicates.

Allele Frequency	Scenario	LoGicAl-pl	LoGicAl-al	LoGicAl-pg	LoGicAl-ag	ASAFE	AFA
(0.001,0.005)	O	0.47	0.12	0.26	0.14	0.71	5.59
	A	0.68	0.13	0.42	0.15	0.72	5.68
	G	0.98	0.23	0.24	0.12	0.72	4.65
	A+G	1.14	0.22	0.30	0.12	0.65	4.15
(0.005,0.01)	O	0.62	0.15	0.36	0.19	0.71	8.59
	A	1.03	0.15	0.61	0.20	0.65	7.92
	G	1.46	0.35	0.35	0.18	0.72	8.30
	A+G	1.85	0.27	0.46	0.17	0.69	6.70
(0.01,0.05)	O	0.78	0.16	0.43	0.21	0.68	8.23
	A	1.11	0.16	0.68	0.21	0.70	6.78
	G	1.29	0.28	0.38	0.19	0.68	8.33
	A+G	1.80	0.24	0.53	0.19	0.66	7.55
(0.05,0.1)	O	0.68	0.16	0.41	0.22	0.73	4.91
	A	0.81	0.15	0.55	0.20	0.67	4.08
	G	1.08	0.25	0.40	0.20	0.70	6.47
	A+G	1.39	0.22	0.53	0.19	0.68	4.91
(0.1,0.5)	O	0.56	0.15	0.40	0.20	0.72	3.40
	A	0.73	0.13	0.47	0.19	0.69	3.06
	G	1.07	0.23	0.41	0.20	0.68	3.41
	A+G	1.24	0.22	0.47	0.19	0.68	3.74

Table S3: CPU runtime (seconds) of ancestry-specific allele frequency estimation methods across allele frequency bins and discordance rates. We simulated $100,000$ phased individuals from a three-way admixture model with inter-continental differentiation ($r = 1$). Methods compared include LoGicAl (LoGicAl-pg, LoGicAl-pg (phased), LoGicAl-ag, LoGicAl-ag (phased)), ASAFE, and AFA under a scenario with probabilistic ancestry calling ($\alpha = 0.1$) and a realistic genotyping error rate ($\theta = 5 \times 10^{-4}$). Runtimes are reported for a single variant and are averaged over 100 simulation replicates.

Allele Frequency	Discordance	LoGicAl-pg	LoGicAl-pg (phased)	LoGicAl-ag	LoGicAl-ag (phased)	ASAFE	AFA
(0.001,0.005)	0	0.42	0.16	0.16	0.04	0.68	7.53
	0.01	0.44	0.16	0.16	0.04	0.69	7.76
	0.1	0.43	0.16	0.15	0.04	0.69	7.81
(0.005,0.01)	0	0.54	0.20	0.18	0.04	0.69	7.90
	0.01	0.56	0.19	0.19	0.04	0.71	7.88
	0.1	0.57	0.19	0.18	0.04	0.68	8.21
(0.01,0.05)	0	0.62	0.22	0.20	0.04	0.70	5.86
	0.01	0.67	0.21	0.21	0.04	0.69	6.11
	0.1	0.61	0.21	0.20	0.04	0.71	6.04
(0.05,0.1)	0	0.58	0.21	0.19	0.04	0.66	4.24
	0.01	0.59	0.23	0.21	0.04	0.70	4.30
	0.1	0.59	0.22	0.21	0.04	0.71	4.29
(0.1,0.5)	0	0.50	0.23	0.19	0.04	0.73	3.04
	0.01	0.52	0.24	0.19	0.04	0.71	2.99
	0.1	0.48	0.22	0.19	0.04	0.70	3.00

Table S4: Allele frequency estimates for LoGicAl-identified strongest ancestry-differentiated variants on chromosome 1 in 1kGP admixed American cohorts.

		LoGicAl			gnomAD			
rsID	Gene Region	AFR	EUR	NA	AFR	EUR	AMR	Ancestry Pair
$rs2814778$	$ACKR1$	> 0.999	0.007	0.005	0.822	0.004	0.090	AFR-EUR
$rs4844412$	$PLXNA2$	0.221	0.108	0.985	0.145	0.151	0.378	NA-EUR
$rs1288600$	$SLC25A3P1$	> 0.999	0.314	< 0.001	0.858	0.273	0.275	NA-AFR

AFR: Sub-Saharan Africa, EUR: Europe, NA: Native America, AMR: Admixed America.

3 Supplementary notes

Note S1: Derivation of LoGicAl algorithm

Following the notations in the Methods section, the complete-data log-likelihood of ancestry-specific allele frequencies is

$$
\begin{aligned}
&l(\boldsymbol{p}_{K\times1}|\boldsymbol{x}_{N\times1},\boldsymbol{\pi}_{N\times2K};\boldsymbol{z}_{N\times2},\boldsymbol{h}_{N\times2}) = \log\Big\{f(\boldsymbol{x}|\boldsymbol{h})\times f(\boldsymbol{h}|\boldsymbol{z};\boldsymbol{p})\times f(\boldsymbol{z}|\boldsymbol{\pi})\Big\}\\
=&\sum_{i=1}^{N}\log\mathbb{Pr}(X_i=x_i|H_i^{(1)}=h_i^{(1)},H_i^{(2)}=h_i^{(2)})+\sum_{i=1}^{N}\sum_{a=1}^{2}\Big(h_i^{(a)}\log p_{z_i^{(a)}}+(1-h_i^{(a)})\log(1-p_{z_i^{(a)}})\Big)+\\
&\sum_{i=1}^{N}\sum_{a=1}^{2}\log\pi_{i,z_i^{(a)}}^{(a)}
\end{aligned}
\tag{1}
$$

The joint conditional distribution of latent variables $(Z_i^{(1)},Z_i^{(2)},H_i^{(1)},H_i^{(2)})$ given the observed data and ancestry-specific allele frequencies is

$$
\begin{aligned}
&w_i(z^{(1)},z^{(2)},h^{(1)},h^{(2)})=\mathbb{Pr}(Z_i^{(1)}=z^{(1)},Z_i^{(2)}=z^{(2)},H_i^{(1)}=h^{(1)},H_i^{(2)}=h^{(2)}|x_i,\boldsymbol{\pi}_i;\boldsymbol{p})\\
=&\frac{\mathbb{Pr}(X_i=x_i|H_i^{(1)}=h^{(1)},H_i^{(2)}=h^{(2)})p_{z^{(1)}}^{h^{(1)}}(1-p_{z^{(1)}})^{1-h^{(1)}}p_{z^{(2)}}^{h^{(2)}}(1-p_{z^{(2)}})^{1-h^{(2)}}\pi_{i,z^{(1)}}^{(1)}\pi_{i,z^{(2)}}^{(2)}}{\sum_{u_1=0}^{1}\sum_{u_2=0}^{1}\sum_{v_1=1}^{K}\sum_{v_2=1}^{K}\mathbb{Pr}(X_i=x_i|H_i^{(1)}=u_1,H_i^{(2)}=u_2)p_{v_1}^{u_1}(1-p_{v_1})^{1-u_1}p_{v_2}^{u_2}(1-p_{v_2})^{1-u_2}\pi_{i,v_1}^{(1)}\pi_{i,v_2}^{(2)}}
\end{aligned}
\tag{2}
$$

Therefore, the expected log-likelihood of ancestry-specific allele frequencies is

$$
\begin{aligned}
&Q(\boldsymbol{p}|\hat{\boldsymbol{p}}^{(t)})=\mathbb{E}[l(\boldsymbol{p}|\boldsymbol{x},\boldsymbol{\pi};\boldsymbol{z},\boldsymbol{h})|\boldsymbol{x},\boldsymbol{\pi};\hat{\boldsymbol{p}}^{(t)}]\\
=&\sum_{i=1}^{N}\sum_{z^{(1)}=1}^{K}\sum_{z^{(2)}=1}^{K}\sum_{h^{(1)}=0}^{1}\sum_{h^{(2)}=0}^{1}w_i^{(t)}(z^{(1)},z^{(2)},h^{(1)},h^{(2)})\Big[\log\mathbb{Pr}(X_i=x_i|H_i^{(1)}=h^{(1)},H_i^{(2)}=h^{(2)})+\\
&\sum_{a=1}^{2}\big(h^{(a)}\log p_{z^{(a)}}+(1-h^{(a)})\log(1-p_{z^{(a)}})\big)+\sum_{a=1}^{2}\log\pi_{i,z^{(a)}}^{(a)}\Big]
\end{aligned}
\tag{3}
$$

Taking derivative for each element p_k in $\boldsymbol{p}$

$$
\begin{aligned}
&\frac{\partial Q(\boldsymbol{p}|\hat{\boldsymbol{p}}^{(t)})}{\partial p_k}\\
=&\sum_{i=1}^{N}\sum_{h^{(1)}=0}^{1}\sum_{h^{(2)}=0}^{1}\frac{\sum_{z^{(2)}=1}^{K}w_i^{(t)}(k,z^{(2)},h^{(1)},h^{(2)})h^{(1)}+\sum_{z^{(1)}=1}^{K}w_i^{(t)}(z^{(1)},k,h^{(1)},h^{(2)})h^{(2)}}{p_k}-\\
&\sum_{i=1}^{N}\sum_{h^{(1)}=0}^{1}\sum_{h^{(2)}=0}^{1}\frac{\sum_{z^{(2)}=1}^{K}w_i^{(t)}(k,z^{(2)},h^{(1)},h^{(2)})(1-h^{(1)})+\sum_{z^{(1)}=1}^{K}w_i^{(t)}(z^{(1)},k,h^{(1)},h^{(2)})(1-h^{(2)})}{1-p_k}
\end{aligned}
\tag{4}
$$

By setting $\frac{\partial Q(\boldsymbol{p}|\hat{\boldsymbol{p}}^{(t)})}{\partial \boldsymbol{p}}=\boldsymbol{0}$, the expected log-likelihood $Q(\boldsymbol{p}|\hat{\boldsymbol{p}}^{(t)})$ is maximized at

$$
\hat{p}_k^{(t+1)}=\frac{\sum_{i=1}^{N}\big[\sum_{h^{(1)}=0}^{1}\sum_{z^{(1)}=1}^{K}w_i^{(t)}(z^{(1)},k,h^{(1)},1)+\sum_{h^{(2)}=0}^{1}\sum_{z^{(2)}=1}^{K}w_i^{(t)}(k,z^{(2)},1,h^{(2)})\big]}{\sum_{i=1}^{N}\sum_{h^{(1)}=0}^{1}\sum_{h^{(2)}=0}^{1}\big[\sum_{z^{(1)}=1}^{K}w_i^{(t)}(z^{(1)},k,h^{(1)},h^{(2)})+\sum_{z^{(2)}=1}^{K}w_i^{(t)}(k,z^{(2)},h^{(1)},h^{(2)})\big]}
\tag{5}
$$

Note S2: Simulation study details

We simulated ancestry-specific allele frequencies following the Balding-Nichols model[1] assuming each ancestral population descends independently from a common reference population. Under this model, the allele frequency p_k of the ancestral population k with reference allele frequency p_A is

$$p_k \sim Beta(p_A \frac{1-F_k}{F_k}, (1-p_A)\frac{1-F_k}{F_k}), k = 1, \dots, K \tag{6}$$

where F_k is Wright's F-statistic[2, 3] measuring the genetic differentiation between the reference population and ancestral population k. Specifically, we simulated reference allele frequency p_A from a uniform distribution $p_A \sim U(a, b)$ with varying (a, b) from $(0.001, 0.005)$ to $(0.1, 0.5)$ to generate allele frequency spectrum from rare to common variants. Based on the simulated p_A, we sampled true ancestry-specific allele frequencies p_k from the distribution (6). Here, to generate ancestral population differentiation, we adopted the design of [4]: we first fixed the total number of ancestral populations at $K = 3$ and set $(F_1, F_2, F_3) = (0.01r, 0.05r, 0.1r)$, where $r \in \{0.2, 0.4, 0.6, 0.8, 1\}$ is a scaling parameter controlling the degrees of population differentiation. This approach has been previously evaluated by [5, 6]. Larger r describes more rapid genetic drift rate from the reference population, making the descending populations more distinct from each other: setting $r = 1$ results in allele frequency differentiation observed between populations across different continents (e.g., between San, Han, and Maya populations), whereas setting $r = 0.2$ corresponds to differentiation between populations on the same continent[5]. Additionally, we varied the number of ancestral populations from $K = 2$ to $K = 5$ and set $F_k = F, k = 1, \dots, K$, with $F = 0.01$ to simulate weak population differentiation and $F = 0.05$ to simulate strong population differentiation.

To simulate true underlying local ancestries and their assignment probabilities, we sampled the assignment probabilities of ancestral groups for each individual independently from a Dirichlet distribution $\boldsymbol{\pi}_i \sim Dir(\alpha \mathbb{1}_K)$, where α quantifies the resolvability of local ancestry inference. We used $\alpha = 0.1$ as the primary setting, based on the estimates from the 1000 Genomes Project cohort (Table S1), and additionally considered $\alpha = 0.5$ to represent lower resolvability. We also generated the assignment probabilities under unbalanced ancestry compositions from asymmetric Dirichlet distributions, $Dir(0.1, 0.1, 0.05)$, $Dir(0.1, 0.1, 0.2)$, and $Dir(0.05, 0.1, 0.2)$. Given the true underlying assignment probabilities $\boldsymbol{\pi}_i$, we further simulated estimated assignment probabilities $\tilde{\boldsymbol{\pi}}_i \sim Dir(\delta \boldsymbol{\pi}_i)$, where δ controls the precision of the estimated probabilities. We varied $\delta \in \{1000, 100, 10\}$: when δ is large, such as $\delta = 1000$, the variability is relatively small and the estimated probabilities are more precise; for example, the standard deviation of $\tilde{\pi}_{i,k}$ is approximately 0.016 when $\pi_{i,k} = 0.5$ and 0.009 when $\pi_{i,k} = 0.1$. When δ is small, such as $\delta = 10$, the corresponding standard deviations increase to approximately 0.151 and 0.090, respectively. Given the simulated true assignment probabilities $\boldsymbol{\pi}_i$, we generated true local ancestry of each allele a from a multinoulli distribution $z_i^{(a)} \sim Multinoulli(\boldsymbol{\pi}_i), a = 1, 2$, while generating the best-guess local ancestry call as the ancestral group where the assignment probability is maximized.

After obtaining the simulated ancestry-specific allele frequencies $\mathbf{p}$ and true local ancestries $\mathbf{z^{(a)}}, a = 1, 2$, we sampled true genotype per chromosome for each individual independently from a Bernoulli distribution $h_i^{(a)} \sim Bernoulli(p_{z_i^{(a)}}), a = 1, 2$. To imitate the effects of genotyping errors and statistical phasing uncertainty, we generated two types of observed genotype inputs: (1) genotype likelihoods representing probabilistic genotype information, and (2) best-guess genotype calls representing discreet genotype assignments.

We generated genotype likelihoods by adopting the framework in [7]. We simulated sequencing coverage for individual i from a Poisson distribution $d_i \sim Poisson(\lambda_i)$ after simulating average sequencing coverage from a truncated normal distribution $\lambda_i \sim TN(\mu, \mu/3), \lambda_i \in (0, 2\mu)$. Here, μ is average sequencing coverage across individuals and we chose $\mu \in \{1, 5, 30\}$ to model low-coverage to high-coverage sequencing data. We then sampled the number of sequencing reads presenting the alternate allele from a Binomial distribution $r_i \sim Bin(d_i, \frac{g_i}{2}(1-\epsilon) + (1-\frac{g_i}{2})\epsilon)$, where $g_i = h_i^{(1)} + h_i^{(2)}$ and ϵ is per-base sequencing error rate. We fixed $\epsilon = 0.001$ across all simulation settings. Therefore, the genotype likelihoods for reference homozygote, heterozygote, and alternate homozygote are $((1-\epsilon)^{d_i - r_i}\epsilon^{r_i}, 0.5^{d_i}, (1-\epsilon)^{r_i}\epsilon^{d_i - r_i})$, respectively. We used simulated overall allele frequency $\bar{p} = \frac{1}{K}\sum_{k=1}^{K} p_k$ as prior and called genotypes for each individual as maximum-a-posteriori estimates based on the posterior probabilities $((1-\bar{p})^2 \times (1-\epsilon)^{d_i - r_i}\epsilon^{r_i}, 2\bar{p}(1-\bar{p}) \times 0.5^{d_i}, \bar{p}^2 \times (1-\epsilon)^{r_i}\epsilon^{d_i - r_i})$.

In parallel, to simulate genotype calls as observed genotype inputs, we generated observed genotype per chromosome from a Bernoulli distribution $o_i^{(a)} \sim Bern(h_i^{(a)}(1-\theta) + (1-h_i^{(a)})\theta), a = 1, 2$, where θ is genotyping error rate and we fixed $\theta = 5 \times 10^{-4}$ as estimated from previous literature[8] to mimic error rates from either array-based (e.g., UK Biobank[9]) or sequence-based (e.g., TOPMed[10]) genotype calling. To simulate observed phased genotypes, we exchanged the values of $o_i^{(1)}$ and $o_i^{(2)}$ with probability γ, generating observed phase $(o_i^{(2)}, o_i^{(1)})$. γ is a compound discordance rate measuring deviations of observed ancestry-genotype phase from true ancestry-genotype phase. These deviations can arise from switch errors and double switch errors (i.e., flip errors) in statistical phasing[8] and inferred ancestry segment flips[11]. Following reported values[12], we varied $\gamma \in \{0, 1\%, 10\%\}$.

References

[1] Balding, D. J. & Nichols, R. A. A method for quantifying differentiation between populations at multi-allelic loci and its implications for investigating identity and paternity. *Genetica* **96**, 3–12 (1995).

[2] Wright, S. Coefficients of inbreeding and relationship. *The American Naturalist* **56**, 330–338 (1922).

[3] Holsinger, K. E. & Weir, B. S. Genetics in geographically structured populations: defining, estimating and interpreting f st. *Nature Reviews Genetics* **10**, 639–650 (2009).

[4] Raj, A., Stephens, M. & Pritchard, J. K. faststructure: variational inference of population structure in large snp data sets. *Genetics* **197**, 573–589 (2014).

[5] Kitada, S., Nakamichi, R. & Kishino, H. Understanding population structure in an evolutionary context: population-specific f st and pairwise f st. *G3* **11**, jkab316 (2021).

[6] Goudet, J. & Weir, B. S. An allele-sharing, moment-based estimator of global, population-specific and population-pair f st under a general model of population structure. *PLoS genetics* **19**, e1010871 (2023).

[7] Meisner, J. & Albrechtsen, A. Inferring population structure and admixture proportions in low-depth ngs data. *Genetics* **210**, 719–731 (2018).

[8] Browning, B. L. & Browning, S. R. Genotype error biases trio-based estimates of haplotype phase accuracy. *The American Journal of Human Genetics* **109**, 1016–1025 (2022).

[9] Bycroft, C. *et al.* The uk biobank resource with deep phenotyping and genomic data. *Nature* **562**, 203–209 (2018).

[10] Taliun, D. *et al.* Sequencing of 53,831 diverse genomes from the nhlbi topmed program. *Nature* **590**, 290–299 (2021).

[11] Atkinson, E. G. *et al.* Tractor uses local ancestry to enable the inclusion of admixed individuals in gwas and to boost power. *Nature genetics* **53**, 195–204 (2021).

[12] Avadhanam, S. & Williams, A. L. Phase-free local ancestry inference mitigates the impact of switch errors on phase-based methods. *G3: Genes, Genomes, Genetics* jkaf122 (2025).
